# Detection of simple and complex de novo mutations with multiple reference sequences

**DOI:** 10.1101/gr.255505.119

**Published:** 2020-08

**Authors:** Kiran V. Garimella, Zamin Iqbal, Michael A. Krause, Susana Campino, Mihir Kekre, Eleanor Drury, Dominic Kwiatkowski, Juliana M. Sá, Thomas E. Wellems, Gil McVean

**Affiliations:** 1 Data Sciences Platform, Broad Institute of MIT and Harvard, Cambridge, Massachusetts 02142, USA;; 2 Wellcome Trust Centre for Human Genetics, University of Oxford, Oxford, Oxfordshire, OX3 7BN, United Kingdom;; 3 Big Data Institute, Li Ka Shing Centre for Health Information and Discovery, University of Oxford, Oxford, Oxfordshire, OX3 7LF, United Kingdom;; 4 European Bioinformatics Institute (EMBL-EBI), Wellcome Genome Campus, Hinxton, Cambridgeshire, CB10 1SD, United Kingdom;; 5 The Wellcome Trust Sanger Institute, Wellcome Genome Campus, Hinxton, Cambridgeshire, CB10 1SA, United Kingdom;; 6 Laboratory of Malaria and Vector Research, National Institute of Allergy and Infectious Diseases, National Institutes of Health, Bethesda, Maryland 20892, USA

## Abstract

The characterization of de novo mutations in regions of high sequence and structural diversity from whole-genome sequencing data remains highly challenging. Complex structural variants tend to arise in regions of high repetitiveness and low complexity, challenging both de novo assembly, in which short reads do not capture the long-range context required for resolution, and mapping approaches, in which improper alignment of reads to a reference genome that is highly diverged from that of the sample can lead to false or partial calls. Long-read technologies can potentially solve such problems but are currently unfeasible to use at scale. Here we present Corticall, a graph-based method that combines the advantages of multiple technologies and prior data sources to detect arbitrary classes of genetic variant. We construct multisample, colored de Bruijn graphs from short-read data for all samples, align long-read–derived haplotypes and multiple reference data sources to restore graph connectivity information, and call variants using graph path-finding algorithms and a model for simultaneous alignment and recombination. We validate and evaluate the approach using extensive simulations and use it to characterize the rate and spectrum of de novo mutation events in 119 progeny from four *Plasmodium falciparum* experimental crosses, using long-read data on the parents to inform reconstructions of the progeny and to detect several known and novel nonallelic homologous recombination events.

High genomic diversity within a population can confound variant and particularly de novo mutation (DNM) discovery efforts. As a single reference genome cannot capture the range of possible haplotypes, short-read aligners assume that new haplotypes are small perturbations to a known canonical reference sequence. Divergent or absent loci violate this assumption; hence, reads sampled from them may align incorrectly or not at all (Landan and Graur 2009). This results in many false positives and false negatives in such regions, the combination of which can sometimes be erroneously interpreted as complex forms of variation. Maps of “genome accessibility” can restrict variant calling to less diverse regions of the genome and reduce such errors (Volkman et al. 2007; Zheng-Bradley et al. 2017; Redmond et al. 2018) but may lead to substantial undiscovered variation.

Of particular concern are de novo structural variants (SVs) driven by mutational mechanisms mediated by microhomology and repeat structure (Carvalho and Lupski 2016). Many SVs are predisposed to occur within repetitive loci around the genome. For example, nonallelic homologous recombination (NAHR) can occur between two low copy-number repeats (LCRs), repetitive sequences ranging from several to hundreds of kilobases in length and having >95% sequence identity between them (Lupski 2004). Nonallelic copies will occasionally be aligned in meiosis and mitosis, with subsequent crossover using them as the substrate for homologous recombination. Resolution of the misaligned sequences can yield successive insertions, deletions, duplications, inversions, and translocations (Parks et al. 2015). NAHR in humans has been associated with several genomic disorders (e.g., Charcot-Marie-Tooth disease type 1A, hereditary neuropathy with liability to pressure palsies) (Lupski 2009) and cancer (e.g., hereditary breast/ovarian cancer) (Xue and He 2014).

For short-read data, SV discovery algorithms examine one or more signals of variation within reads aligned to a canonical reference sequence. These signals include paired-end (PE) read analysis (i.e., clusters of read pairs with significantly different insert sizes or orientations than expected), changes in read depth (RD), identification of split reads (SRs) and/or soft-clipped (SC) reads (for a comprehensive overview, see Cameron et al. 2019). Reads showing such signals are then either examined directly or used to construct a local assembly of the putative SVs. Among the best-performing germline SV detection algorithms are DELLY (Rausch et al. 2012), GRIDSS (Cameron et al. 2017), and Manta (Chen et al. 2016). DELLY uses PE and SR evidence to characterize deletions, inversions, tandem duplications, and translocations but does not identify insertions or events <300 bp in length. GRIDSS is a local assembly method, first extracting reference-aligned reads with putative evidence for a variant (SR/SC reads as well as discordant PE reads), assembling the selected reads, and aligning the resulting contig back to the reference sequence in order to identify SVs. Manta similarly identifies SR/SC/PE reads and constructs a breakend association graph whose edges denote long-range adjacencies. Reads associated to individual edges are then assembled and aligned to the reference genome to facilitate SV identification.

Many SV detection algorithms use a heuristic cutoff on putative event length to avoid processing the entirety of the genome and focus their computational efforts on the most plausible SV candidates and thus are insensitive by design to variation below a preset threshold (typically 50 bp). For variant characterization below this threshold, additional tools (particularly for SNVs and small indels) must be applied. The GATK HaplotypeCaller (Poplin et al. 2017) tool examines mismatch and indel signals in reference-aligned reads to identify intervals (“active regions”) up to 300 bp in length that may harbor variation. These reads are then assembled into candidate haplotypes that are scored by the maximum likelihood estimate (MLE) of the pair-HMM alignment of the original input reads to the candidates. Alignments of the haplotype to the reference are then parsed for variant candidates, and the base quality scores and per-read haplotype likelihoods are used to calculate the posterior probability of genotypes for each variant. Downstream filtering based on RD, read mapping quality, strand bias, and other indicators of error are applied to reject likely false-positive calls.

Local assembly around candidate variants is efficient but inherently biased toward the reference sequence. Whole-genome de novo coassembly of short-read data provides a means for overcoming reference bias, capturing a more comprehensive account of variation and facilitating a direct comparison among samples (Iqbal et al. 2012). However, the repetitive nature of many SVs precludes the straightforward application of existing tools (Alkan et al. 2011; Tattini et al. 2015). A typical assembly graph stores genomic subsequences *k*-mers as vertices and sequence overlaps (read-to-read alignments or *k* − 1 substring matches) as edges (Flicek and Birney 2009). Repeats longer than the vertex length collapse into a single copy. Differing sequence contexts manifest as multiple edges, which is problematic for assembly as extracting unambiguous contiguous sequence from a graph requires runs of vertices with an in-degree and out-degree of one (“unitigs”).

For small sample sizes, de novo assembly using long-read data from third-generation sequencing is a viable strategy for overcoming reference bias and assembling through highly repetitive loci (Rhoads and Au 2015; Jain et al. 2018). However, the high-molecular-weight gDNA input requirement relative to second-generation sequencing (∼5000 ng vs. ∼1 ng) is difficult to satisfy with some samples. Many pathogens grow slowly in culture, requiring several months or even years to expand to sufficient amounts for long-read sequencing. Stromal contamination and high heterogeneity in cancer samples compromises the ability to acquire pure samples of such high mass, and amplification risks PCR replication artifacts masquerading as true DNMs.

Instead, it may be possible to (1) leverage the relative strengths of both short- and long-read data, (2) examine multiple related samples for variation simultaneously, and (3) overcome reference bias by comparing the samples’ genomes directly. Consider a scenario in which one sequences a small number of samples with long reads to augment a larger, short-read data set. For a typical assembly from short-read data (e.g., 76-bp reads, >20× coverage), sequencing is expected to recover nearly (i.e., barring systematic sequencing errors and ultra-low-complexity sequences that fail to amplify) every *k*-mer in the genome (Lander and Waterman 1988), even if the reads do not provide sufficient genomic context to navigate through repetitive regions. That context can be provided by aligning long haplotypes to the short-read graph, annotating edge choices, and following these choices when traversing the graph (Turner et al. 2018). These long haplotypes need not be from the sample itself; recent common ancestry among samples leads to extensive sharing of variation that can be used to guide assembly in related samples. By demanding that the short-read genome graph is immutable (after initial construction and removal or correction of likely sequencing errors), the process of long-haplotype alignment cannot add any new vertices and can only provide connectivity information through existing vertices. This naturally constrains the alignments to informing connectivity in regions of high (but not necessarily perfect) homology between the long-read and short-read samples. Finally, by aligning multiple data sets to the graph (many long-read data sets, PE reads from the sample itself, etc.), we can assemble through recombination breakpoints by transitioning between annotation sets. In essence, rather than using existing tools to improve accuracy of long-read assemblies with short reads (Koren et al. 2012; Salmela and Rivals 2014; Walker et al. 2014; Goodwin et al. 2015), we improve the connectivity of short-read assemblies with long reads.

## Results

### Missing genomic novelty in reference-based analysis

**Figure 1.**
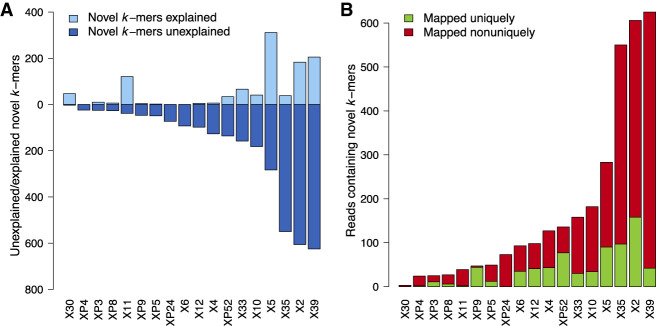
Extent of reference-characterized and -uncharacterized novelty among 18 progeny from an experimental cross between 3D_7_ and HB_3_ *P. falciparum* isolates, sequenced by the MalariaGen project (Illumina 76**-**bp reads, ∼100× coverage). (*A*) Novel *k*-mers observed in the reference-based analysis (“explained”; bars *above x*-axis) versus novel *k*-mers remaining from the reference-free analysis (“unexplained”; bars *below x*-axis). (*B*) Reads that map uniquely to the reference genome (MQ > 0; green) versus mapping multiple times or not mapping at all (*MQ* = 0; red), conditioned on the read containing a novel *k*-mer. For further details, see Supplemental Material.

We motivate the development of a reference-unbiased DNM discovery tool by first exploring discrepancies in genomic novelty identified by reference-based versus reference-free analyses. We examined 20 high-coverage *Plasmodium falciparum* samples, the etiological agent of malaria, from the MalariaGen project's sequencing of crosses between two substantially diverged parasites (Miles et al. 2016). We compared a conservative list (strong filtering) of novel sequences present in short-read de novo assemblies of progeny versus a liberal list (no filtering) of novel sequences from haplotypes combinatorially produced from multiple reference-based variant callsets on the same data. This comparison is shown in Figure 1A (for further information on comparison procedure, see Supplemental Fig. S1). If a reference-based callset captured all sequence diversity in a sample, our expectation is that all novel sequences would be captured by variant calls and thus “explained.” However, even with strong filtering on the de novo assembly data and no filtering on the reference-based callset data, 28% ± 22% (min = 0%, max = 94%) of novel sequences in the assemblies did not correspond to any reference-based variant call. Most of these unexplained novel “*k*-mers” (length *k* substrings from reads) were found in reads that failed to map or that mapped nonuniquely to the reference sequence (Fig. 1B).

### A de novo coassembly approach to DNM discovery

**Figure 2.**
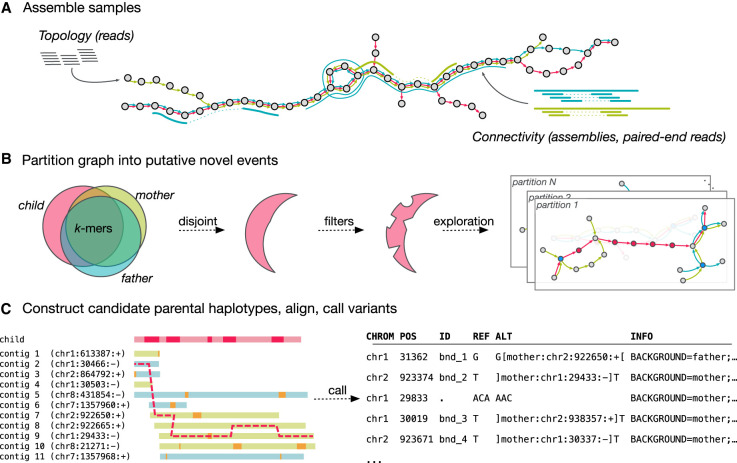
Overview of the Corticall algorithm. (*A*) Samples are assembled into a multicolor linked de Bruijn graph (LdBG). Short, accurate reads are used to determine graph topology. Longer sequences derived from paired-end reads or from draft/finished assemblies are thread through the graph, providing information on connectivity to overcome repeats but not adding novel *k*-mers. (*B*) Novel *k*-mers, sequences present in the progeny and absent in the parents, are filtered and then used to signal the presence of putative de novo mutations (DNMs). Subgraphs around such events are extracted, forming a set of variant candidates. (*C*) Regions flanking novel *k*-mers are assembled to reveal candidate parental haplotypes. The progeny's contig is probabilistically aligned to the set of candidate parental contigs, allowing for mismatches, indels, and (potentially nonallelic) recombination. The resulting alignment thus specifies parental background and (if reference sequences are available) coordinate information. Variants (SNVs, MNVs, indels, translocation breakends, etc.) within the novel *k*-mer regions are returned as likely DNMs.

To overcome limitations in the reference-based analysis described above, we developed a DNM discovery approach consisting of three steps. First, de novo assembly, based on multicolor linked de Bruijn graphs (LdBGs; described below), is used to store and link adjacent *k*-mers for each sample. These assemblies are error-cleaned; that is, low-frequency *k*-mers likely to be the result of sequencing errors are removed from the graphs. Unlike error correction, error cleaning does not add new (and potentially unobserved) sequence to the graph. Second, trusted “novel” *k-*mers are identified, which are sequences unique to the individual progeny, indicative of DNMs, that are unlikely to arise from error or contamination. Finally, novel *k*-mer-spanning contigs are aligned to reconstructed sequences in the parents, identifying the nature of the event that generated the DNM. Figure 2 depicts these steps, detailed in the Supplemental Material and summarized below.

### Connectivity preserved in multicolor LdBGs

We have previously reported on multisample and multicolor de Bruijn graphs (dBGs) for straightforward reference-free genome comparison between multiple samples (Iqbal et al. 2012) and LdBGs for improved assembly via read-to-graph and reference-to-graph alignment annotations (Turner et al. 2018). Briefly, an LdBG is a multigraph (Zwillinger 2011) representation of multiple genomes that preserves “stackability” (easy comparison of multiple samples via inner joins by *k*-mer of per-sample coverage and edge information) and connectivity information inherent in reads and/or long input haplotypes. As illustrated in Figure 2A, input reads are decomposed into *k*-mers and stored as graph vertices. Each sample is assigned a unique identifier (or “color”). Colored edges are placed between vertices representing *k* − 1 overlaps with another *k*-mer in the same sample. Reads and/or haplotype data (e.g., alternate reference assemblies) are then aligned to the graph (once per color) by trivial lookups of shared *k*-mers. Discrepancies between the sequence and the graph manifest as missing *k*-mers, correctable by traversing the graph between the gap boundaries or truncating the alignment if the correction attempt fails. At junctions (vertices with in-degree or out-degree greater than one), the edge consistent with the aligned sequence is recorded in an auxiliary file. All junctions spanned by an alignment are annotated with relevant link information, ensuring traversal can begin anywhere in the graph and still have access to complete navigation data. During traversal, we collect links in the order they are encountered, assigning each link an “age” reflecting the number of vertices traversed since collected and using the oldest link to specify junction choices. If a conflict arises between multiple oldest links, we halt traversal.

### Novel *k*-mers are signposts for DNMs

We build upon this genome comparison framework by first identifying regions of the joint pedigree graph (an LdBG containing sequence data for parents, progeny, and optional reference sequences) to explore for potential DNMs. As such mutations are by definition present in the progeny and absent in the parents, *k*-mers spanning these events would also be expected to be exclusive to the progeny.

An accurate list of novel *k*-mers serves both as an indicator of DNM presence around the graph and a measure of how many mutational events are available for discovery. However, iteration over the graph and selection of putative novel *k*-mers (those with zero coverage in the parents and more than zero coverage in the progeny) will yield a set enriched for sequencing errors and other artifacts that obscure the small fraction of *k*-mers arising from genuine DNMs. We apply multiple filters to remove such artifacts (specifically, contamination; graph tips; low-complexity sequence; “orphans,” sequence found in the progeny but with no edges to parental sequences; low-coverage *k*-mers; “unanchored” *k*-mers, *k*-mers in branches that have no unique alignment in any provided genome; and *k*-mers shared by other progeny; for details, see Methods). We verified these filters by examining novelty in simulated *P. falciparum* crosses and a real trio for which we obtained Pacific Biosciences (PacBio) sequencing on both parents and progeny.

### Contigs spanning novel *k*-mers contain putative de novo events

Next, we “partition” the graph into subgraphs, grouping novel *k*-mers into separate bins based on their proximity to one another within the graph. This is illustrated in Figure 2B. Each partition may harbor one or more DNMs, but DNMs are not split across multiple partitions. At each novel *k*-mer, we walk along the progeny's color in the pedigree graph, exploring outward and constructing the longest possible contigs. To maximize contig length (and thus increase our sensitivity to complex variation), we use two strategies. First, links derived from haplotype alignments (e.g., draft references, PE reads, etc.) are used to disambiguate junction choices. Second, as DNMs will typically yield a succession of novel *k*-mers in a graph and as the previous filtering step will have removed most artifacts, we walk past junctions when one (and only one) of the outgoing edges at a novel *k*-mer connects to another novel *k*-mer. This procedure, which we have termed “novel *k*-mer aggregation,” ensures that proximate novel *k-*mers are considered together, useful for large SVs that may manifest as a series of nearby, but nonadjacent, runs of novel *k*-mers.

### Assembling adjacent parental contigs for event decoding

We then construct parental sequences that constitute the candidate haplotypic background(s) for a DNM. At each parentally shared *k*-mer in a partition, we initiate a contig assembly in the parents. The presence of novel *k*-mers in the partition may lead to gaps in the parental contigs not automatically filled by this assembly step. We close these gaps via depth-first searches (DFSs) between bordering *k*-mers. To prevent a combinatoric explosion of considered paths, we limit our explorations to depths of 1000 bp by default. For gaps we fail to close in this manner, we assemble flanking boundaries up to a maximum of 500 bp.

Each contig is given a label specifying the parental background from which it was reconstructed and a unique index. If draft/finished reference sequence data are available, we additionally attach coordinate information by aligning each parental contig to the associated draft reference sequence via a built-in version of BWA-MEM (BWA-MEM Java bindings developed by Pierre Lindenbaum, https://github.com/lindenb/jbwa) (Li 2013).

### “Mosaic” alignment reveals simple and complex mutations

To identify mutations, determine parental background, and assign genomic coordinates, we apply a pair-HMM to simultaneously align and phase progeny contigs over candidate parental haplotypes. This model, originally used to study evolutionary relationships in a set of highly diverse antigenic genes from the *P. falciparum var* gene family (Zilversmit et al. 2013), combines the probabilistic models for sequence alignment (Durbin 1998) and the detection of recombination events (for trellis diagram of model, see Supplemental Fig. S3; for model parameter definitions, see Supplemental Table S7; Li and Stephens 2003). Recast in a SV framework, it enables simultaneous discovery of both simple/complex mutations in a panel of sequences that are not prealigned to one another. As our model permits recombination between any site and any candidate parental haplotype, it also enables the detection of nonallelic events, such as NAHR.

Briefly, the method is as follows. Consider a query sequence (the contig in the progeny) and a set of *N* source sequences (contigs in both parents, partially or completely spanning the target sequence). Our goal is to describe the target sequence as a set of match/mismatch, insertion, deletion, and recombination operations on the source sequences. We choose the starting point in the source sequence uniformly across all sites in the source sequences, beginning in the match or insert states with some probability. At each position, there exists the probability of jumping to any target sequence and any position via recombination. The maximum likelihood alignment (and trajectory through the target panel sequence space) is obtained using the Viterbi algorithm. Variant calls are obtained by examining the traceback path and identifying differences with respect to the query sequence. This process is depicted in Figure 2C (an expanded representation on a similar toy sample is presented in Supplemental Fig. S4).

A simple set of postprocessing filters are applied to keep false-discovery rates low. For all mutational types, we reject events containing fewer than five novel *k*-mers. We additionally require NAHR events to satisfy one of two conditions: (1) Multiple breakends are detected within a single contig, and (2) single breakends are detected within 2000 bp of breakends satisfying (1).

### Simulation: novel *k*-mer detection and increased contig lengths

To evaluate our ability to correctly detect DNMs in assembly data, we generated an in silico pedigree of 1000 progeny. This was accomplished in two stages: (1) simulation of full-length (23-Mb) haploid genome sequences for each progeny sample and (2) simulation of reads for each genome sequence. For each genome sequence, we incorporated a wide range of de novo events for later evaluation. Annotated draft reference sequences constructed for two *P. falciparum* isolates (HB3 and DD2) (see Supplemental Material, section S2) were used as parental genomes. We computed *k*-mer–based homology maps per sister chromatid and modeled crossovers per chromosome based on empirical rates provided in Miles et al. (2016; for simulated map lengths and per-chromosome crossover probabilities, see Supplemental Fig. S5), keeping track of the relocated members of each parent's *var* gene repertoire. We then added simple and complex DNMs, simulating small (1- to 100-bp), intermediate (101- to 500-bp), and large (501- to 1000-bp) events and placing them randomly throughout the genome. In addition to simulating SNVs, MNVs/indels with random sequence, and inversions, special care was taken to simulate variants arising from repeat expansion and contraction by searching for existing repetitive regions in the genome and adding or subtracting repeat units. NAHR events were simulated by recombining members of the progeny's *var* gene repertoire after meiotic recombination. Assuming a low DNM rate, three random events were simulated per progeny.

To generate reads for these synthetic genomes, we simulated 76-bp PE reads with an insert size distribution of 250 ± 50 bp, stochastic coverage of 100×, and a sequencing error rate of 0.5% (∼*Q*23). These values were comparable to existing data on the HB3×DD2 cross (Wellems et al. 1990, 1991; Miles et al. 2016). We constructed joint pedigree graphs using existing Illumina data for the HB3×DD2 parents along with our simulated reads for the progeny, applying the assembly procedure detailed in Supplemental Material, section S3.5 (initial assembly at *k* = 47, error cleaning, and PE read and draft reference threading), and extracted novel *k*-mers according to the procedure in Methods.

**Figure 3.**
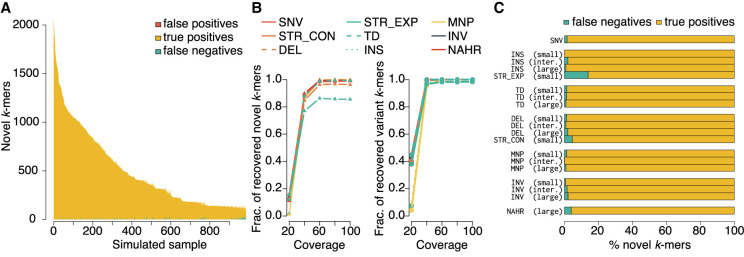
Simulation-based evaluation of novel *k*-mer detection and subsequent reassembly quality for contigs spanning novel *k*-mers in error-containing short-read data. (*A*) Number of *k*-mers in the progeny correctly identified as novel (true positives), undetected (false negatives), and misidentified as novel (false positives). (*B*) Novel and variant *k*-mer recovery for all in silico progeny at simulated mean coverages of 20×, 40×, 60×, 80×, and 100×. (*C*) For all simulated alleles, the fraction assembled completely (i.e., wholly contained within a single contig) and incompletely (i.e., only partially reconstructed).

We first evaluated our novel *k*-mer detection procedure on these simulated data sets. Figure 3A summarizes our detection of true and false novel *k*-mers. We were able to detect 90.0% ± 22.7% of expected novel *k*-mers per sample. Novel *k*-mers that we failed to detect were typically low-complexity or repetitive sequences (generated de novo by the mutation process but also occurring elsewhere in the genome). For these events, a *k*-mer size of 47 bp was insufficient to resolve the sequences as novel.

Next, we examined the recovery of novel and variant *k*-mers as a function of short-read coverage and by downsampling coverage on the simulated genomes to values between 20× and 100×. As coverage increases, the fraction of expected novel *k*-mers increases, saturating at ∼60× (Fig. 3B, left panel). However, not all novel *k*-mers generated by a mutational event need be recovered to tag a variant. Despite some novel *k*-mers being lost to filtration, enough remain such that effective variant reconstruction can still occur at ∼40× average genome coverage (Fig. 3B, right panel).

Finally, we sought to more clearly understand the relationship between missed novel *k-*mers versus the type and length of variant event from which they arose, summarized in Figure 3C. Across all variant types, 97.8% ± 3.1% of novel *k*-mers generated by mutational events are detected. The bottom three performers are short tandem repeat (STR) contractions, STR expansions, and NAHR events, in which the percentage of novel *k*-mers detected are 86.1%, 95.4%, and 96.0% respectively. This is to be expected; all three mutational classes are manipulations of repetitive sequence, the expansion/contraction/recombination of which would be plausibly expected to generate *k*-mers already present in other repeats in the genome.

### Simulation: mutation detection and evaluation

We applied Corticall and four other variant calling software packages (DELLY, GRIDSS, HaplotypeCaller, and Manta) to the simulated set of 1000 HB3×DD2 progeny. Although our software is specifically designed to leverage multiple reference sequences and identify variants on the closest haplotypic background, the latter four algorithms are not. Attempting to characterize variation in simulated HB3 and DD2 haplotypes absent from the 3D7 reference does not provide an effective demonstration of the other algorithms’ capabilities. Instead, we developed a procedure to run each alternate caller twice on child reads aligned to the HB3 and DD2 reference genomes separately, integrate the resulting callsets (taking care to exclude redundant variants appearing in syntenic regions of the parental genomes), and filter out inherited and likely false-positive mutations. We quantified caller performance by computing *F*_1_ scores for different variant classes, requiring type compatibility, 80% reciprocal overlap of alleles, and correct parental background identification. We also computed a more lenient *F*_1_ score wherein these requirements were significantly relaxed. Further details are provided in our Supplemental Material. The aggregated results for all simulated samples are shown in Table 1.

**Table 1.**
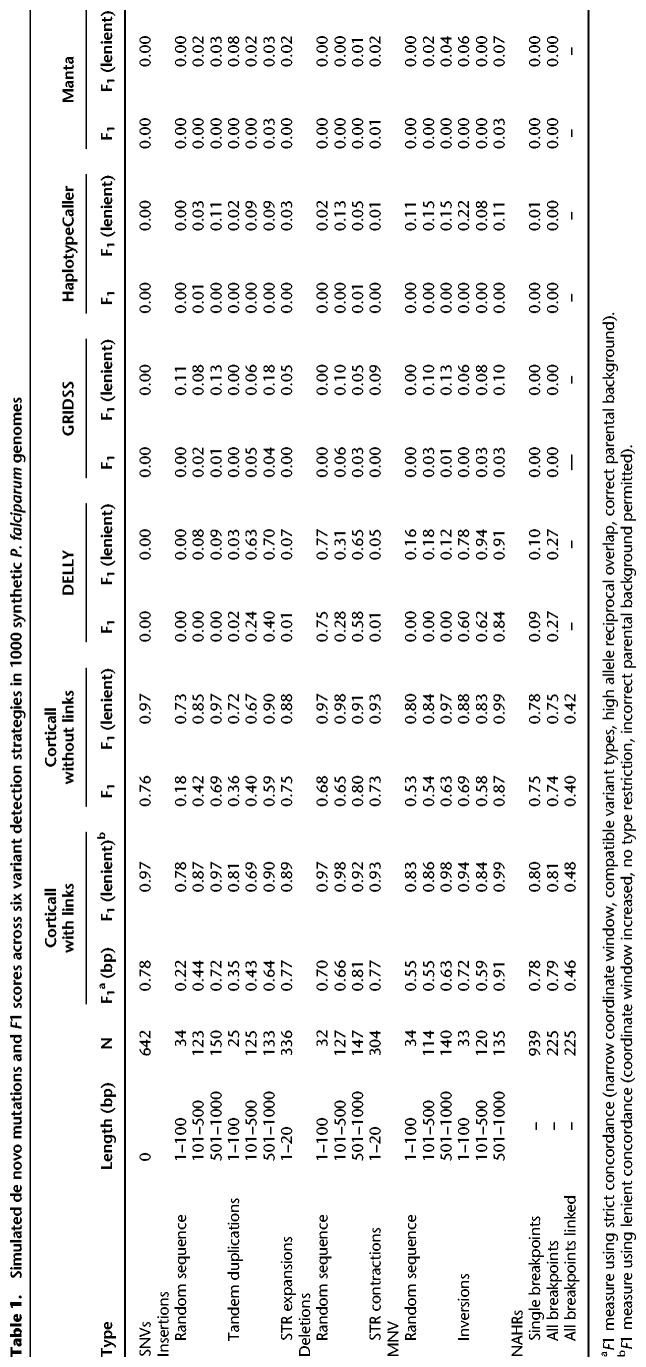
Simulated de novo mutations and *F*1 scores across six variant detection strategies in 1000 synthetic *P. falciparum* genomes

Overall, we found that >90% of detected novel *k*-mers are assignable to variant events, and >86% of simulated variants are identified (either partially or completely reconstructed). This changes very little with assembly mode as, aside from some light filtering, the absence or presence of link information does not alter the detection of novel *k*-mers. Instead, it simply alters the number of contigs into which a variant assembles. For complete reconstruction of each variant event, *F*_1_ uniformly increases between the link-uninformed and link-informed reconstruction as link information provides a means to overcome repetitive regions of the assembly.

We measured calling performance on NAHR breakends and further our ability to detect all breakends within a single event. Although both unlinked and linked reconstructions are generally able to detect the presence of a breakend, the reconstructions with links show a marked improvement in event characterization. This permits multiple breakends to be observed on a single contig, enabling detection and assignment of all breakends within a NAHR event to a single call and simplifying variant classification. As the other tested variant callers do not specify which variants were found on the same contig, this metric could not be assessed for other algorithms.

Corticall substantially outperformed other algorithms in the detection of DNMs across all variant classes except inversions. Of the alternate algorithms evaluated, DELLY provided results most comparable to our own, performing particularly well on tandem duplications, deletions, and inversions (outperforming Corticall for 101- to 500-bp inversions) but, by design, did not identify short indels and multinucleotide variants (MNVs). DELLY was also able to detect more NAHR breakends than any alternate algorithm except our own. Given that these events were simulated in noncore *var* genes having little homology between the HB3 and DD2 repertoires, recovery performance was unaffected by relaxing the restriction that events be identified on the correct haplotypic background.

The other SV detection methods (GRIDSS and Manta), and SNV/small indel caller (HaplotypeCaller) underperformed considerably at DNM detection compared with Corticall and DELLY. Although poor sensitivity was a substantial issue for these approaches, these low *F*1 measures are more attributable to the difficulty in controlling the high false-positive rate, even after filtering out inherited variation and syntenic sites and after applying a battery of depth, mapping quality, and strand bias filters. This could potentially be remedied by using a similar novel *k*-mer approach to Corticall, permuting the reference sequence with putative variants to identify spanning *k*-mers and variants with *k*-mer support in the parental read data sets.

### Core, noncore, and repetitive region DNM detection in *P. falciparum*

To characterize the number and type of DNMs occurring in the genome of the malaria parasite *P. falciparum*, we applied our software to sequencing data from four *P. falciparum* experimental crosses: 3D7×HB3 (Walliker et al. 1987), HB3×DD2 (Wellems et al. 1990), 7G8×GB4 (Hayton et al. 2008), and 803×GB4 (Sá et al. 2018). Seeking to obtain finished or draft reference sequences for all parents in the crosses, we first obtained the canonical 3D7 reference genome from PlasmoDB (Gardner et al. 2002). We additionally obtained recently generated high-quality draft reference assemblies for the HB3, DD2, and 7G8 parental genomes. Finally, we generated new PacBio draft assemblies for the GB4 and 803 parental genomes, as well as one progeny genome from the 803×GB4 cross (36F11). Except for the 3D7 reference sequence, all assemblies were made using PacBio RSII sequencing data (∼100×, 10- to 15-kbp per sample). We verified our long-read assembly procedure by comparing the canonical reference sequence to our version of the 3D7 genome (for a dotplot comparison between the two assemblies, see Supplemental Fig. S2). NCBI accessions for new GB4, 803, and 36F11 genomes are provided in “Data access” and ERA accessions for all PacBio data sets are provided in Supplemental Table S1. Further data set and assembly metrics are provided in Supplemental Table S2.

**Figure 4.**
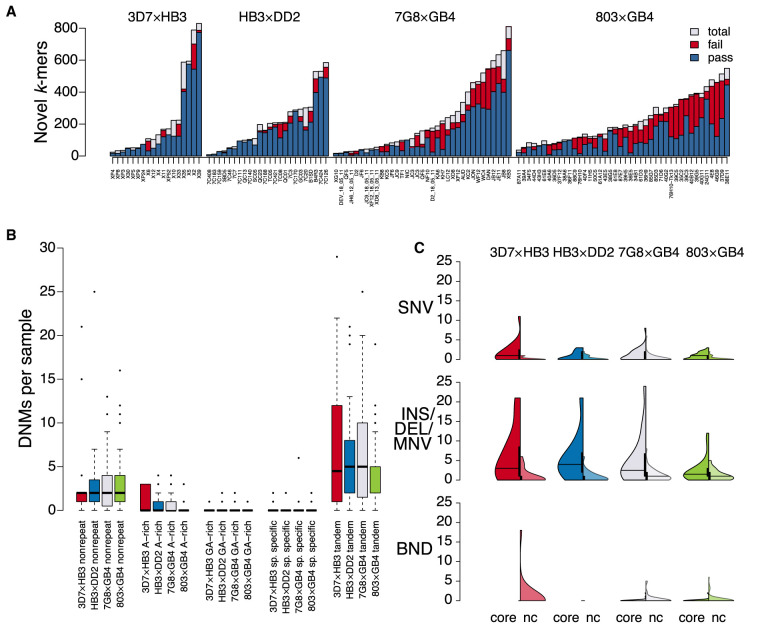
Per-sample DNM discovery metrics in 119 *P. falciparum* progeny. (*A*) Novel *k*-mers per cross and sample (gray bars). For those contained within successfully assembled variants, *k-*mers in variant passing filters are shown in green; the rest are shown in red. (*B*) Per-cross DNM sample distributions for mutations appearing in repetitive regions of the respective parental genomes. (*C*) Violin plots showing DNM sample distributions per cross, split by those in core genomic regions (*left*) and noncore regions (*right*).

We then obtained Illumina data for all parents and progeny in the experimental crosses (NCBI accessions in Supplemental Table S3; metrics in Supplemental Table S4) and generated McCortex assemblies at *k* = 47. After contaminant and outlier removal, we called DNMs in 119 progeny and verified our calling procedure on real data by manually comparing novel *k-*mers and variant calls between the Illumina and PacBio data sets for the 36F11 progeny parasite (Supplemental Tables S5, S6, respectively). Calls across all four crosses are summarized in Figure 4.

Across samples, we assigned putative variant calls to 89% ± 11% of novel *k*-mers (Fig. 4A). Their impact was greatest in the 803×GB4 cross, where the 803 and GB4 draft reference sequences have comparatively poorer assembly qualities of *Q*28 and *Q*23, respectively (compared with *Q*28 and *Q*29 for 3D7 and HB3). After filtering, we detected a total of 972 DNMs (163 SNVs, 348 insertions, 322 deletions, 19 MNVs, seven NAHR events, and 113 incompletely assembled events). The average per sample DNM count is low, with short indels (approximately 5.47 per sample) outnumbering SNVs (approximately 1.29 per sample).

To determine the functional effect of each variant, we transferred existing 3D7 gene models and performed ab initio gene prediction on each parental genome via the Companion (Steinbiss et al. 2016) annotation server. We then concatenated gene models for each cross and annotated all variants with SnpEff (Cingolani et al. 2012). Taking the first (most deleterious effect) listed, these results are summarized in Table 2. As expected, the majority of events (∼85%) landed outside of gene coding regions, including nearly all incompletely assembled variants (likely owing to the difficulty of assembling the low-complexity and repetitive intergenic loci). Relatively few missense or conservative in-frame mutations are observed (7%), and even fewer stop-gain, frameshift, or otherwise disruptive indels (3%) are detected.

**Table 2.**
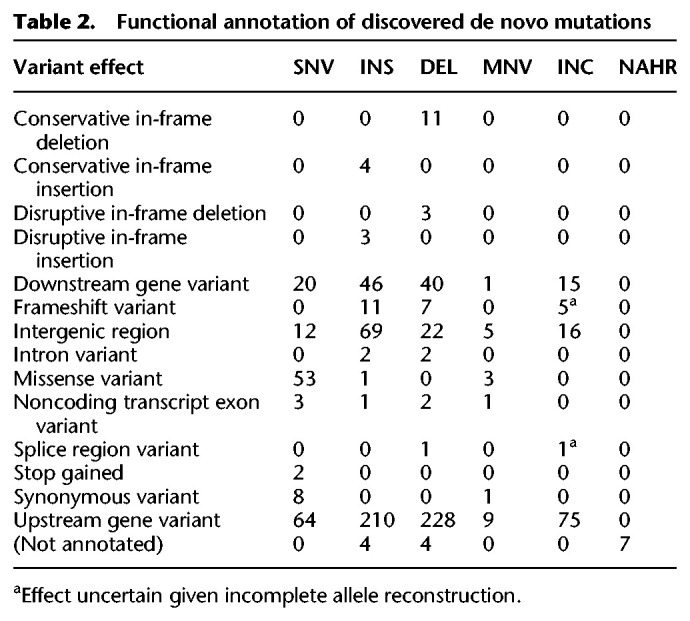
Functional annotation of discovered de novo mutations

We applied the RepeatMasker (Smit et al. 2013) software to annotate repetitive genomic sequences and Spine (Ozer et al. 2014) to annotate noncore genomic regions (sequences private to each parasite isolate, typically encompassing subtelomeric/hypervariable regions) across all parental genomes. We then inspected variant locations with respect to these annotations (Fig. 4B,C). Aggregated across all samples and crosses, we found a threefold enrichment of mutations occurring in repetitive genomic regions, ∼90% of which fell within tandem duplications. Mutations were enriched in the noncore (∼2 Mb) versus core (∼21 Mb) genomic compartments (SNVs: *P* = 1.5 × 10^−7^; INS/DEL/MNV: *P* < 2.2 × 10^−16^; BND: *P* < 2.2 × 10^−16^; based on chi-squared tests accounting for indel lengths and number of novel *k*-mers appearing in NAHR events) (see Supplemental Material). We observed similar per-sample mutation distributions across samples.

We computed per-sample per-nucleotide mutation rates across all four crosses. Additionally, as DNMs can continue to accumulate in each parasite during the in vitro intraerythrocytic lifecycle, we computed mutational rates per nucleotide and generation. However, culture time and lifecycle time for cross progeny was not always known. Assuming a culture time of 52 d between initial cloning and sequencing (the average of the documented culture times for the 3D7×HB3 and HB3×DD2 cross progeny), and a mitotic generation time of 48 h (Trampuz et al. 2003), Per-nucleotide mutational rates are presented in Table 3. These rates are broadly consistent across crosses and compartments and with previous estimates based on parasite clone trees (Bopp et al. 2013; Claessens et al. 2014).

**Table 3.**
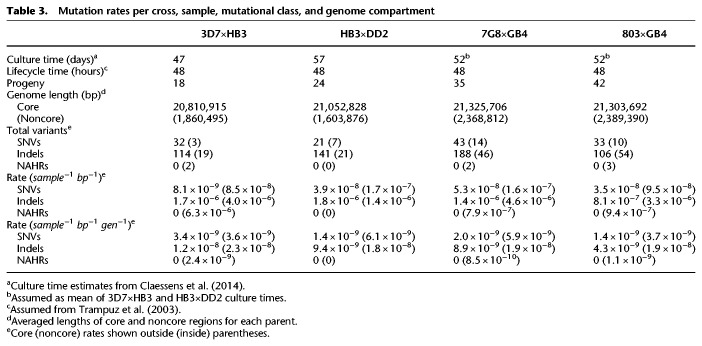
Mutation rates per cross, sample, mutational class, and genome compartment

### Hypothesis-free discovery of NAHR events at base-pair resolution

**Figure 5.**
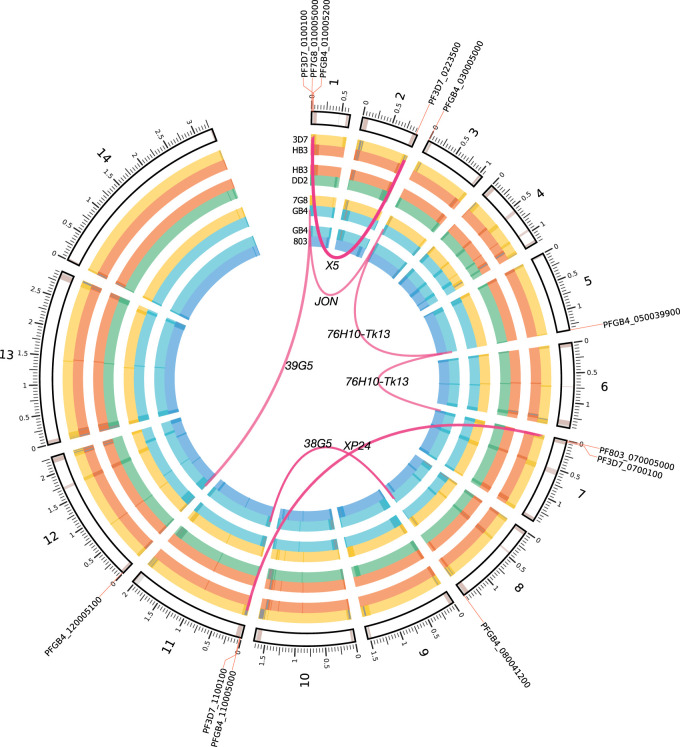
Circos (Krzywinski et al. 2009) plot of NAHR events detected in all 119 samples across four *P. falciparum* experimental crosses. Parental genomes for each cross are depicted in the inner grouped circular tracks. Bézier curves depict each translocation event, with termini indicating the parent(s) of origin and a label at the apex of the curve identifying the sample in which it was found. Closest gene names annotated on outer circumference. Dark bands indicate noncore regions determined by the Spine (Ozer et al. 2014) software, except in the outer ideogram, which is based on alignability maps for the canonical 3D7 reference genome (Miles et al. 2016).

To detect NAHR events, we grouped proximate breakend calls and applied three filtration criteria: (1) Events must contain 20 or more novel *k*-mers, (2) events must consist of three or more breakends, and (3) at least one contig must link distal genomic loci within the same contig. We detected seven NAHR events in total after filtration, depicted in Figure 5. All occurred in subtelomeric noncore regions of the genomes. Examining these events with respect to our new gene models, all but four of the genes closest to a breakend were related to antigenic gene families and immune evasion.

Previous work on NAHR events in *P. falciparum*—based on observations of apparent translocations of *var* gene sequences and limited by inadequate reference sequences for parasites other than 3D7—have only reported NAHR events within the exon 1 of *var*-gene family members (Deitsch et al. 1999; Freitas-Junior et al. 2000; Frank et al. 2008; Duffy et al. 2009; Bopp et al. 2013; Claessens et al. 2014; Sander et al. 2014). As we enforce no a priori hypothesis on which loci are likely to harbor such recombinations, the discovered events in our data set extend beyond *var* exon 1. We summarize these events in Table 4. Although the events still occur in the subtelomeric regions of the genome (within which many other genes related to immune evasion reside), five of the 12 genes proximate to the NAHR breakends were not *var* genes. A single event occurred near a gene from the *rif* gene family.

**Table 4.**
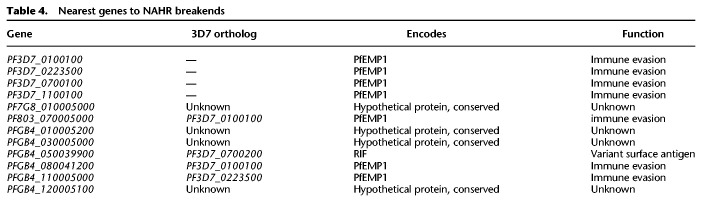
Nearest genes to NAHR breakends

**Figure 6.**
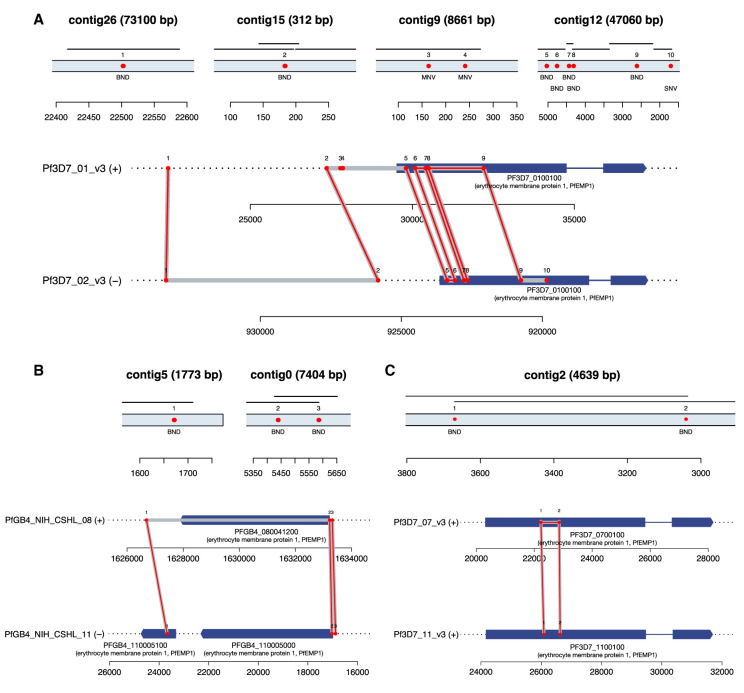
Three of the detected NAHR events in the *P. falciparum* crosses. (*A*) NAHR event involving two *var* genes in 3D7×HB3 progeny X5 (*PF3D7_0100100* on Chr 1, *PF3D7_0223500* on Chr 2). (*Top*) LdBG contigs spanning mutation (dBG contig shown as thin black line for comparison). Called mutations shown along contig as red points. (*Bottom*) Mutations from LdBG contigs in genomic context shown in red. Gene models shown in dark blue (thick lines: exonic sequence; thin lines: intronic sequence). Inferred recombination path shown in gray. (*B*) NAHR event in 803×GB4 sample 38G5 (*PFGB4_080041200* on Chr 8; *PFGB4_11005100* and *PFGB4_11005000* on Chr 11). (*C*) NAHR event in 3D7×HB3 sample XP24 (*PF3D7_0700100* on Chr 7 and *PF3D7_1100100* on Chr 11).

Beyond identifying new NAHR events outside of the usual *var*-gene repertoire, we were also able to clarify the extent of previously observed events. Figure 6 depicts three of the detected NAHR events. In Figure 6A, our calls recapitulate previously reported rearrangements (breakends 5–9) within the long exons of *PF3D7_0100100* and *PF3D7_0223500* (*PFA0005w* and *PFB1055c* in older nomenclature) (Sander et al. 2014). Flanking these known breakends are a number of mutations that have not been previously reported, including an additional series of breakends upstream of each of the *var* genes (1 and 2), two MNVs (3 and 4), and an SNV within the coding region of the antigenic gene on Chromosome 2. In panel B, a novel NAHR event is shown with a recombination path that weaves in and out of coding regions, touching upon the previously unexamined exon 2. The recombination path within the novel event in panel C (within a sample in the previously unpublished 803×GB4 cross) remains wholly within the coding sequence.

Among the isogenic pair 76H10 and 76H10-Tk13 (a transformant clone C580Y allele in the Kelch propeller domain of K13) (see the section *pfk13* Modification in the supplemental information of Sá et al. 2018), the latter shows multiple recombination breakpoints absent in the untransformed counterpart. It is also the only parasite in the data set to show breakends linking the subtelomeric ends of more than two chromosomes. Further inspection revealed the 310 novel *k*-mers underlying these events to be unique to 76H10-Tk13 and unlikely to be a filtering artifact; zero overlap was observed between the 310 novel *k*-mers present in the transformed clone (76H10-Tk13) after filtering and the 42,549 novel *k*-mers present in the untransformed clone (76H10) before the application of filters. 76H10-Tk13 required several months of continuous cultivation, including transfection with zinc-finger plasmids to induce allelic substitution and subsequent limited cloning dilution. The presence of these breakends in the transformed clone and their absence in the untransformed clone suggests the acquisition of structural mutations in long-term continuous culture. This finding comports with the similar observations by Bopp et al. (2013) indicating substantial telomeric plasticity relative to the core region of the genome in *P. falciparum* parasites propagated in vitro for 180 generations.

### Variant calling with cumulatively expanding reference set

Exploring beyond comparisons of progeny-to-progenitor genomes, we hypothesized that genomic novelty present in a sample but not placeable on the background of an evolutionarily distant reference sequence would be better elucidated through the simultaneous use of multiple reference sequences. We obtained Illumina data and constructed a PacBio draft assembly of an 803×GB4 progeny (36F11). From the 36F11 data, we extracted *k*-mers that were novel with respect to the 3D7 reference genome, further filtering these *k*-mers based on presence in the counterpart 36F11 clone assembly, thus constructing a conservative *k*-mer list that flags true variation in the 36F11 parasite. We used this list to seed variant calls, increasing the number of reference sequences provided with each callset.

**Figure 7.**
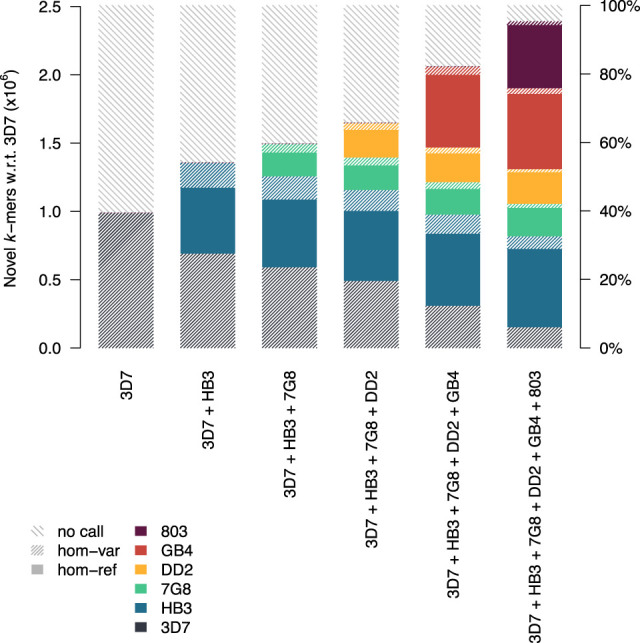
Calls tagged by 36F11 *k*-mers novel with respect to 3D7, redescribed against combinations of other reference sequences. Stacked bars represent fraction of novel *k*-mers linked to homozygous-reference (hom-ref) and homozygous-variant (hom-var) calls or of *k*-mers where no call could be made. Colors represent the specific haplotypic background the call was placed on (if a call can be equally described on multiple backgrounds, one is chosen at random).

Figure 7 depicts the calling results on 36F11 with the cumulative addition of 3D7, HB3, 7G8, DD2, GB4, and 803 reference sequences. As novel *k*-mers are computed with respect to 3D7, calls at these *k*-mers can only be homozygous-variant. As additional reference sequences are added, variants are described against a new background sequence. However, many novel *k*-mers tagging variation against 3D7 are no longer considered novel with respect to another reference sequence, and their reconstructed sequence for the progeny is perfectly homologous to the additional reference. Thus, as more reference sequences are added, apparent variation against 3D7 is redescribed as homozygous-variant (hashed bars) or homozygous-reference to a sequence other than 3D7 (solid bars). When using all six reference sequences, our ability to characterize apparent novelty to 3D7 grows from 40% to 95%.

## Discussion

We have presented a graph-based DNM calling method, available through our software Corticall, that is capable of discovering simple and complex variants in pedigrees and experimental crosses without bias toward a reference sequence. Our approach leverages long-haplotype data derived from any source (existing finished genomes, draft assemblies from third-generation sequencing, targeted sequencing of specific loci, etc.) to improve the assemblies of other short-read data sets. These long-haplotype samples need not be from the same sample. Short-read data are used to establish graph topology, whereas long-haplotype data are aligned to the graph but constrained to specify connectivity information only. Sequencing errors (and possibly mutations) are always adjudicated in favor of the existing graph; thus, no new sequence is added, only navigation information. This approach opens the opportunity for multiple long-read data sets to be used to improve the connectivity of many more short-read assemblies.

Corticall can leverage many finished or draft reference-quality data sets, seamlessly transitioning between connectivity information sets during assembly. This affords a powerful approach to the hypothesis-free study of DNMs. As many of these events occur in repetitive or genetically diverse regions of the genome, the use of multiple reference sequences during assembly helps to provide access to so-called genomic “dark matter,” loci underserved by pure short-read de novo assembly or a single canonical reference.

Corticall assembles variants, not genomes, and keeps false-discovery rates low by only inspecting regions of the genome harboring novel *k*-mers. By combining local, multisample assembly with a simultaneous alignment/recombination model, we are able to detect a wide variety of mutational types with a single, consistent framework. Additionally, tracking the number of novel *k*-mers explained by each variant call provides a useful metric for determining the completeness of the final callset.

In the *P. falciparum* crosses, we detected SNVs at rates broadly consistent with previous work, as well as indels at more than four times the SNV rate. We detected new NAHR events, all in subtelomeric regions of the genome that are not represented in the canonical reference. For previously discovered NAHR events, we are able to find additional breakends in nearby noncoding regions, establishing a more complete picture of nonallelic recombination behavior in these pathogens. Much of the de novo mutational spectrum appears in noncore regions. These compartments are diverse in the population precisely because they typically harbor clinically relevant genes underlying drug resistance or immune escape functionality. The mapping-free, reference-agnostic approach espoused by Corticall thus enables the detection of this clinically relevant variation and removes the requirement for determining the appropriate genome reference for mapping and analysis.

The fixed record size structure of Cortex graphs used with Corticall enables storage in an ordered, randomly accessible manner, thus keeping memory requirements low as the entire graph need not be loaded into memory in order to be inspected. Predetermining the novel *k-*mers to inspect, along with intelligent caching to prevent redundant lookups when assembling multiple samples over shared *k*-mers, reduces disk accesses. As a result, Corticall is able to scale to genomes of any size. This may provide a valuable approach to the study of Mendelian disease in large pedigrees or tumor/normal pairs (wherein the normal can be considered as the parent of the tumor samples).

Corticall has several limitations, addressable by future work. Although Corticall need not load an entire graph into memory to perform variant calling, the genome assembly software upon which it relies does require that the entire graph be stored in RAM as it is being constructed. Thus, even though the variant calling step on human data can be performed in as little as 1 GB of RAM, the initial de novo assembly step still requires hundreds of gigabytes of memory to execute. Recent approaches to streaming graph construction (Rozov et al. 2018) and/or succinct dBGs (Conway and Bromage 2011; Muggli et al. 2017) may well address this limitation.

Additionally, our use of long-haplotype data is restricted to sequences that have been substantially error-corrected. Typically, *k*-mer sizes used in dBG-based short-read assemblies (e.g., *k* = 31–96) are still too high for the long, error-prone reads generated by third-generation sequencers. However, lowering the *k*-mer size of the short-read assemblies to a length more likely to result in a perfect match on the long-read data (e.g., *k* = 11) would result in too many junctions from homologous sequences in the graph. Our current approach to error-correcting long reads against the graph requires that the path through the existing graph contain no junctions, and would thus be impaired by setting the *k*-mer size too low. A more computationally expensive read-to-graph alignment procedure could remedy this limitation.

Finally, the generalizability of Corticall to more mutational types and diploid/polyploid organisms can be improved in the future by expanding our signals for putative variation beyond novel *k*-mers. Novel *k*-mers restrict our search for putative variation but may limit our sensitivity to some classes of variation. Large copy number variants, inversions, and mobile element insertions typically rearrange or reorient existing sequences in the genome and thus may not always give rise to novel *k*-mers. Instead, their presence would be signaled by changes in coverage (Nijkamp et al. 2012) and/or patterns of graph connectivity (Lemaitre et al. 2014), which can be found by appropriate comparison between coassembled samples. Future work should capture these variant types by additionally considering *k*-mer coverage and graph motifs.

With the introduction of the high-yield PacBio Sequel II platform with circular consensus (or “HiFi”) sequencing, as well as continued innovation in base-calling by Oxford Nanopore to lower the per-read error rate, the construction of additional draft reference genomes is becoming more accessible. The utility of these data extends beyond pure de novo assembly for constructing new reference sequences or for elucidating structural variation in single samples. Strategic choices as to which samples to sequence with long reads can enable simple and complex variant discovery in a much larger cohort while simultaneously keeping costs low provided that variant calling methods are capable of leveraging such information. Corticall is a step forward in this direction, presenting a uniform approach to variant discovery and typing that combines assembly, alignment, recombination models, and third-generation reference sequence panels. Such approaches will assist in overcoming bias to a single canonical reference sequence and enable a more complete description of variation in diverse populations.

## Methods

### Assembly of long-read data

We performed PacBio RSII sequencing to ∼100× coverage (per vendor recommendation) on DNA from the six experimental cross parents (3D7, HB3, DD2, 7G8, GB4, 803) and a single progeny clone from the 803×GB4 cross, 36F11. We performed de novo assembly on each isolate using HGAP2/HGAP3; removed potential sequence contaminants; performed pseudochromosome contiguation (to facilitate easy comparison with the canonical reference sequence for isolate 3D7), and annotate gene, repeat, and core/noncore genome compartments. Further details of sample preparation, sequencing, assembly, annotation, quality assessment, and download links are provided in the Supplemental Material.

### Assembly of short-read data

We analyzed data from 119 individual isolates from four *P. falciparum* experimental crosses collected and sequenced in the MalariaGen Genetic Crosses project (https://www.malariagen.net/projects/p-falciparum-genetic-crosses). Isolates were sequenced using the Illumina GAII or HiSeq platforms to obtain PE reads ranging from 76 to 100 bp with a target coverage of ≥100×. We performed de novo assembly on each isolate using the McCortex (Turner et al. 2018) assembler, using the aforementioned long-read assemblies to augment the connectivity of each short-read genome graph. Further details of sample preparation, sequencing, assembly, and download links are provided in the Supplemental Material.

### Overview of the Corticall algorithm

Our DNM calling strategy is based on identifying mutational motifs in a “multicolor LdBG” (Iqbal et al. 2012; Turner et al. 2018). This can be decomposed into three steps. First, we construct LdBGs from short-read and long-haplotype data sets. Second, for each so-called “novel” *k*-mer (those unique to a child and absent from its parents), we assemble a child contig and one or more parental contigs containing *k*-mers shared with the child contig. Finally, we perform probabilistic all-to-all alignment allowing for recombination, attempting to describe the child's sequence as a series of match, insertion, deletion, and recombination operations on a panel of candidate parental sequences. Decoding the traceback of the probabilistic alignment yields variant calls. Details on each step are provided below.

### Construction of the LdBG

Briefly, a dBG for sample *c* is formulated as a set of vertices and edges, $G_{c}=\{Vc,Ec\}$. Vertices Vc are input sequences are broken into fixed length substrings of length *k* (“*k*-mers”) with unit stride, and edges Ec encode *k* − 1 overlaps of adjacent vertices. Each record is recorded as three columns: a *k*-mer sequence, its coverage, and its incoming/outgoing edges. *N* sample graphs constructed at identical *k* can be “stacked” by performing a full (outer) join on *k*-mer sequences, each sample *c*’s coverage and edge information simply being recorded as two additional columns in each *k*-mer record. Stacking facilitates easy comparison of the graphs of *N* samples and formally yields a union graph $G={⋃}_{c=1}^{N}G_{c}$. This formulation encodes relationships between two adjacent *k*-mers (the *i*th and (*i* + 1)-th *k*-mers in a sequence, as well as the (*i* − 1)th and *i*th), but relationships between nonadjacent *k*-mers are lost. Thus, even if an input sequence spans a repeat when a single *k*-mer does not, the connectivity information inherent in the sequence is not retained. We restore this connectivity by trivially aligning input sequences $R_{c,d}$ from data set *d* to graph $G_{C}$. The addition of new vertices to the graph during alignment is disallowed; the process merely amounts to lookups of shared *k*-mers between the input sequence and the graph and to bridging gaps over sequence differences with simple walks on $G_{C}$. For all junctions (vertices with in-degree or out-degree greater than one) spanned by an input sequence, we record the series of disambiguating edge choices (referred to as “links”), exhaustively annotating all participating junctions with relevant navigation information. We refer to this composite data structure (graph and links) as a LdBG, $G=\left\{V_{c},E_{c},{⋃}_{d=1}^{D}L_{c,d}\right\}$, where $L_{c,d}$ is a sparse set of links on graph color *c* derived from sequence data set *d*.

### Using links during LdBG navigation

By exhaustively annotating all spanned junctions with links, we ensure that traversal initiated anywhere in the graph has access to complete link information. Upon initiating a walk at vertex *vc*, we collect each link we encounter. At a junction, we consult our list and extract the oldest link (i.e., the link that was obtained earliest in the traversal), as this link establishes the greatest context as to location in the genome. If there are multiple links with the same age that disagree as to the next junction choice, we halt traversal.

### Identification and filtration of novel *k*-mers

In a multicolor dBG representing parents and children from a pedigree or an experimental cross, the locations of most DNMs will be signaled by the presence of novel *k*-mers: sequences unique to a child's genome and absent from both parental genomes. The set of novel *k*-mers in a child should also provide an indication as to how much novelty in a genome remains to be explained by some mutational process. As sequencing errors and sample contamination will also contribute to the set of novel *k*-mers, we sought to identify all novel *k*-mers in a child's graph and remove potential errors and contaminants. We identified and developed filters for five common graph or sequence motifs indicative of error:

1. **Contamination.** Contamination presents as a subset of novel *k*-mers that are unique to the sequencing data for a child but are irrelevant to the study at hand. To remove these sequences, each entry in the initial set of putative novel *k*-mers was screened for contamination via BLAST (Altschul et al. 1990). We rejected any *k*-mer with a match of any quality to an organism other than the species under study. To account for mutations present in our contaminants but absent in the BLAST database, we used the contaminating *k*-mers as starting points for DFSs in our graphs, exploring the child's graph until it rejoins a parent's graph and rejecting all *k*-mers along the way.
2. **Graph tips.** Graph tips present as a series of novel *k*-mers that bifurcate from a parental graph but never rejoin. They are typically the result of sequencing errors at the ends of reads but could also reflect true variation and subsequent coverage drop-out during sequencing. However, in the latter case, such variation tagged by novel *k*-mers would still not be recoverable without further sequencing data to fill in the missing coverage. To remove graph tips, we perform DFS from a putative novel *k*-mer, expecting to rejoin a parental graph on both ends. If exploration on one end connects to a parent and fails on the other end, we reject all child *k*-mers contained in the traversal.
3. **Promiscuously connected sequences.** Low-complexity sequence (or “dust”) may manifest as *k*-mers promiscuously connected to many other low-complexity *k*-mers, presenting as an unnavigable graphical tangle. We defined such dust *k*-mers as those having a sum of in-degrees and out-degrees greater than four. We initiated DFS at such *k*-mers, exploring until we either run out of edges to navigate or rejoin a parental graph and keeping track of the number of *k*-mers traversed since the last time we observed one of low complexity. If we reach one of the aforementioned stopping conditions and the distance traversed since the last low-complexity *k*-mer is less than the graph's *k*-mer size, we consider the traversed vertices to be dust and reject all elements.
4. **Highly compressible sequence.** Additional low-complexity sequences are detected by computing the compression ratio (“CR”) of the *k*-mer (gzip-compressed length vs. uncompressed length) and removing any putative novel *k*-mer with a CR less than a predefined threshold (by default 0.703 for 47-bp *k*-mers).
5. **Orphans.** Graphical orphans are a series of novel *k*-mers that fail to ever connect to a parental graph. They may include contaminants absent from the BLAST database or reads with unusually high sequencing error. We performed DFS at putative novel *k*-mers, rejecting *k*-mers from traversals that joined one of the parental colors at any time.

We also removed putative novel *k*-mers from consideration based on two additional criteria:

1. **Shared *k*-mers.** Putative novel *k*-mers, although absent from parents, may be shared among children. Some of these may reflect recurrent DNMs, but the overwhelming majority stem from recurrent sequencing errors. We remove *k*-mers shared with other children (omitting clones of a child from consideration).
2. **Low coverage.** A number of putative novel *k*-mers substantially less than the mean coverage of the sample. Such *k*-mers may still permit navigation to flanking regions with coordinates in a parental genome, despite arising from sequencing error. We remove *k*-mers with coverage less than a specified value (by default, 6×).

The bulk of sequences captured by these final two filters are likely to be recurrent sequencing error. However, we note that they could also remove a small number of DNMs from our consideration.

### Query sequence assembly

To construct sequences spanning putative variants, we perform contig assembly at each novel *k*-mer on the query sample (e.g., the child). Unless otherwise specified, these assemblies are conducted using McCortex links generated by threading the sample's PE read data and the parental assembly data through the query sample's graph (Turner et al. 2018). Optionally during graph traversal, if we encounter a junction vertex that (1) is itself a novel *k*-mer and (2) cannot be traversed with links and if (3) one (and only one) of the outgoing vertices is also a novel *k*-mer, then we assume both novel *k*-mers are part of the same mutational event and extend contig construction through these vertices. As assemblies seeded by proximate novel *k*-mers may result in redundant contigs, we postprocess the contig set to remove redundant sequences and those fully contained by other contigs. Finally, if multiple contigs share a novel *k*-mer, we remove all but the contig that contains the largest number of novel *k*-mers. This effectively “partitions” the contig set into those representing distinct mutational events.

### Source sequence assembly

For each query sequence, we build a panel of source sequences to which the query is aligned. At each nonnovel *k*-mer in the query sequence, we perform contig assembly on the source samples (e.g., the parents). Unless otherwise specified, these assemblies are conducted using McCortex links generated by threading the sample's PE read data and the parental assembly data through the child's graph. During assembly, gaps at the boundaries of mutational events in the query sample may be incompletely assembled owing to sequencing error or graph homology. We close these gaps via DFS between gap boundaries. If still not closed, we assemble gap flanks by a maximum of 500 bp. Flanking sequence irrelevant to the query is trimmed by subsetting the source within the boundaries of the earliest and latest *k*-mers shared with the query sequence.

Each source sequence is given a unique label, simply incrementing from first to last. If a reference sequence is specified for the relevant sample in the LdBG, the source sequence is aligned to that reference using BWA-MEM and relabeled with the resulting genomic coordinates. Note that the relabeling step does not alter the source sequence in any way.

### Variant typing by simultaneous alignment to reference genome panels

Two general classes of graphical variant motifs concern us: “bubbles” (SNVs, short indels and inversions, multinucleotide polymorphisms) and “breakends” (large indels and inversions, NAHRs, gene conversions, and allelic recombinations). We address both classes of variants in a single probabilistic framework wherein a novel *k*-mer-spanning contig (“query” sequence) is simultaneously aligned to a panel of candidate haplotypes (“source” sequences). We achieve this by repurposing the Tesserae model (Supplemental Material; Zilversmit et al. 2013), a pair-HMM combining models for global alignment with affine gap penalty (described by Durbin 1998) and haplotype diversity estimation via recombination (Li and Stephens 2003), to the task of bubble and breakpoint variant typing. Briefly, we assume a query sequence arises as an imperfect mosaic of source sequences. For each query and its candidate source sequences (collectively referred to as the “sequence set,” *h*), we apply the Viterbi algorithm to find the maximum likelihood path through our pair-HMM.

The model (including formal descriptions of the Viterbi, forward, and backward algorithms) is fully specified by Zilversmit et al. (2013). The pair-HMM is specified by a transition matrix and emission matrix, detailed in the Supplemental Material.

## Data access

The GB4, 803, and 36F11 PacBio sequencing generated in this study have been submitted to the NCBI BioProject database (https://www.ncbi.nlm.nih.gov/bioproject/) under accession number PRJEB31043.

Source code and a precompiled release of Corticall is provided as Supplemental Code and is freely available at GitHub (https://github.com/mcveanlab/Corticall). This software is released under the open-source Apache 2.0 license.

## Competing interest statement

G.M. is a founder and director of Genomics PLC and a partner in Peptide Groove LLP.

## Supplementary Material

Supplemental Material
