# Supplementary material for: Detection of simple and complex de novo mutations with multiple reference sequences
Source: Genome Res. 2020 Aug;30(8):1154–69. doi: 10.1101/gr.255505.119 (PMC7462078; doi:10.1101/gr.255505.119)
Supplement: Supplemental Material [file supp_30_8_1154__index.html]

Detection of simple and complex de novo mutations with multiple reference sequences — Supplemental Material 

# Detection of simple and complex de novo mutations with multiple reference sequences

## Supplemental Material

- Supplemental\_Material.pdf
- Supplemental\_Code.tar.zip
