# Supplementary material for: Detection of simple and complex de novo mutations with multiple reference sequences
Source: Genome Res. 2020 Aug;30(8):1154–69. doi: 10.1101/gr.255505.119 (PMC7462078; doi:10.1101/gr.255505.119)
Supplement: Supplemental Material [file supp_gr.255505.119_Supplemental_Code.tar.zip › Supplemental_Code.tar.gz!/Corticall-0.5/manuscript/dnm_preprint_supp.pdf]

Supplemental Material

Kiran V Garimella[1,2,3], Zamin Iqbal[2,4], Michael A. Krause[2,5,6], Susana Campino[5], Mihir Kekre[5], Eleanor Drury[5], Dominic Kwiatkowski[3,5], Juliana M. Sa[6], Thomas E. Wellems[6], and Gil McVean[2,3]

[1]Data Sciences Platform, Broad Institute of MIT and Harvard
[2]Wellcome Trust Centre for Human Genetics, University of Oxford
[3]Big Data Institute, Li Ka Shing Centre for Health Information and Discovery, University of Oxford
[4]European Bioinformatics Institute (EMBL-EBI), Wellcome Genome Campus
[5]The Wellcome Trust Sanger Institute, Wellcome Genome Campus
[6]Laboratory of Malaria and Vector Research, National Institute of Allergy and Infectious Diseases, National Institutes of Health

Table of Contents

Introduction 2
S1 Missing novelty in reference-based scans for *de novo* mutations 4
S1.1 Reference-free novel sequences 4
S1.2 Reference-based novel sequences 4
S1.3 Comparison 6
S2 Methods for DNM discovery based on linked genome assembly graphs 7
S2.1 Overview 7
S2.2 Construction of the linked de Bruijn graph 7
S2.3 Using links during LdBG navigation 7
S2.4 Identification and filtration of novel *k*-mers 8
S2.5 Query sequence assembly 9
S2.6 Source sequence assembly 9
S2.7 Variant typing by simultaneous alignment to reference genome panels 9
S3 Data processing: long-read sequencing, assembly, and alignment 13
S3.1 Data availability 13
S3.2 Library preparation 13
S3.3 *De novo* assembly 13
S3.4 Contaminant removal 14
S3.5 Pseudochromosome contiguation and gene annotation 14
S3.6 Annotation of repetitive regions and accessory compartments 14
S3.7 Quality assessment 14
S4 Data processing: short-read sequencing, alignment, and assembly 17
S4.1 Data availability 20
S4.2 Library preparation 20
S4.3 Alignment 20
S4.4 Reference-based variant calls 20
S4.5 *De novo* assembly 21
S5 Simulation of progeny genomes 21
S5.1 Homologous recombination 21

S5.2 Non-homologous allelic recombination 22
S5.3 Other variants 22
S5.4 Read simulation 23
S5.5 Evaluation 23
*

Introduction

Here, we provide further detail on the motivation, methods, data acquisition, processing, and analysis steps underlying the results presented in this work. Software, manuscript source, and additional resources (e.g. genome assemblies, machine-readable text files with paths to data downloads) are available at the following locations:

Purpose	URL	License
Genome assembly	https://github.com/mcveanlab/mccortex	MIT
Variant assembly	https://github.com/mcveanlab/CortexJDK	Apache 2.0
Additional resources	https://github.com/mcveanlab/CortexJDK/tree/master/manuscript	-

S1 Missing novelty in reference-based scans for *de novo* mutations

Our main manuscript presents an analysis wherein genomic novelty in several *P. falciparum* isolates is quantified using two separate methods and compared. This analysis is detailed below.

Consider a scenario in which genomic sequence sampled from an individual is absent or sufficiently disparate from a canonical reference genome. Many sequenced reads may fail to align to the reference correctly. Variants spanned by those reads may thus be rendered undiscoverable by reference-based approaches to variant detection. However, quantifying this missingness — the number of *de novo* mutations (DNMs) in a sample that remain to been discovered — is not straightforward.

We hypothesized that this missingness could be quantified by comparing a reference-*free* DNM novel sequence set (i.e. one constructed by performing *de novo* assembly on all members of the pedigree and emitting haplotype sequences unique to the children) to a reference-*based* novel sequence set (i.e. one constructed by aligning pedigree reads to a reference sequence, identifying mutations, and emitting haplotype sequences implied by those mutations). In an ideal scenario where divergence between samples and the reference is very low, the reference-free and reference-based analyses should be equivalent. With real data, the lack of equivalence can provide insight into how much novelty within a sample remains to be identified.

We performed such an analysis on *P. falciparum* progeny from the 3D7xHB3 experimental cross (20 progeny, 76-bp reads, $\sim$100x coverage, see Table S5 for further detail). The analysis workflow follows and is additionally diagrammed for clarity in Figure S1.

S1.1 Reference-free novel sequences

DNMs can be considered generators of novel genomic sequence — sequence that is present in an individual's genome but absent from their parents. Rather than counting mutational events explicitly, we instead count sequences unique to the genome of the child. We used the McCortex software(27) to split sequenced reads into fixed-length subsequences of length $k = 47$ ("k-mers"), aggressively removed sequencing errors and other artifacts using McCortex's k-mer-frequency-based cleaning and additional filters described in section S2.4, and compute the disjoint between child's k-mers and parental k-mers (Figure S1a-c). This provides a conservative reference- and alignment-free method of computing the *total* amount of novelty in a child's genome.

S1.2 Reference-based novel sequences

We aligned parental and progeny reads to the canonical *P. falciparum* reference of the 3D7 isolate using `bwa mem`(12), called SNVs and small indels using HaplotypeCaller(20) (GATK v4.0.2.1 with the `-ploidy 1` setting) and larger indels and structural variants using Delly(21) (v0.7.8). We did not apply any filtration steps in an effort to generate a callset with maximum sensitivity.

To generate k-mers that could be compared with the reference-free analysis, we sought to permute the reference sequence with mutations from the callset and emit k-mers spanning the mutations. Given the high false discovery rate of the unfiltered callset, we could not rely on a single haplotype with all variants incorporated. Instead, we generated nearly all possible variant haplotypes within small genomic windows. At each putative mutation, we collected the previous 50-bp of reference sequence and a maximum of 10 variants within the immediately following 50-bp window. We combinatorically constructed all haplotypes from every possible subset of

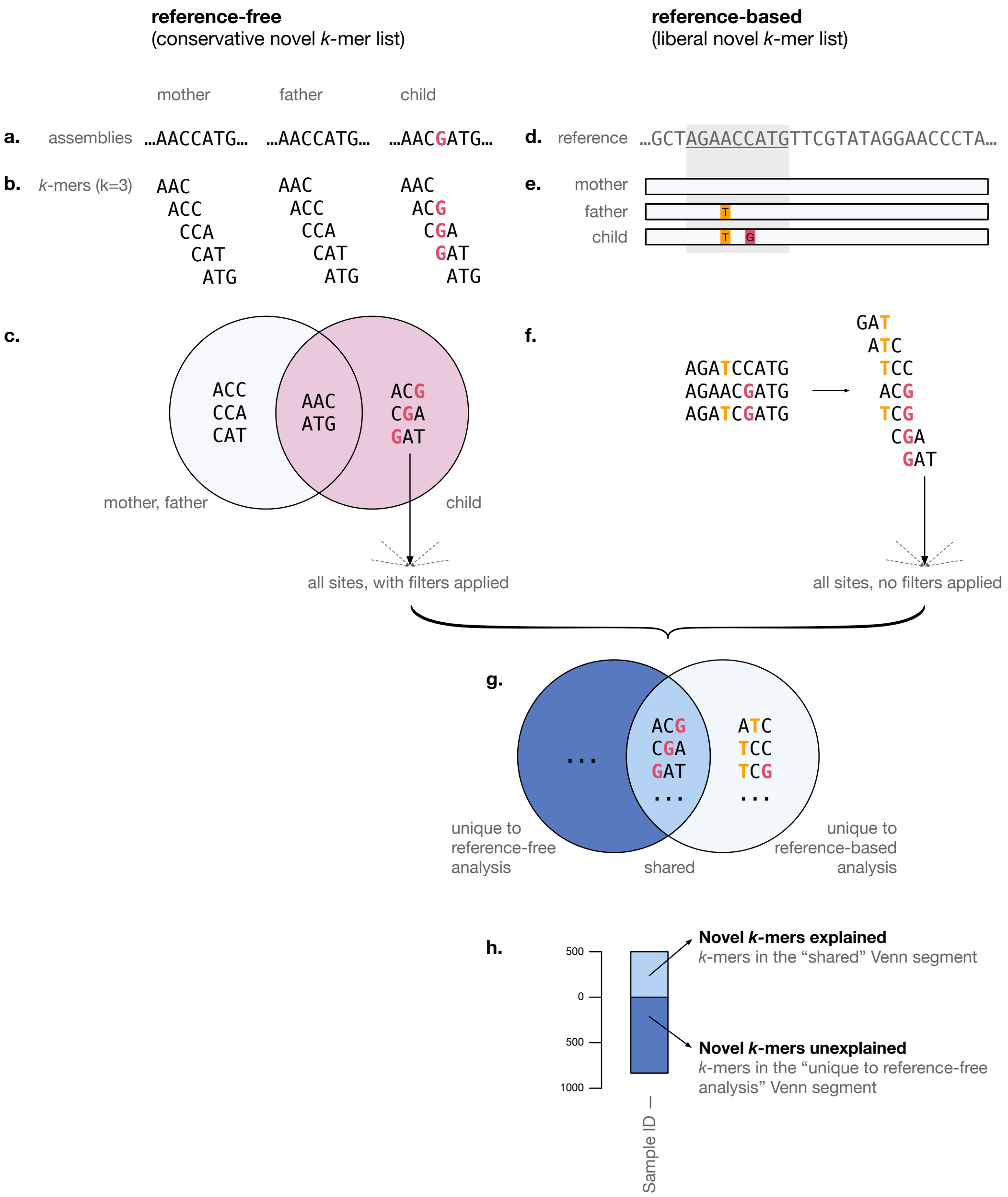

Figure S1. Procedure for comparing reference-free and reference-based scans for genomic novelty. *Reference-free pipeline:* (**a**) Reads are assembled *de novo* and error-cleaned into a multi-color de Bruijn graphs using the McCortex software. (**b**) Error-cleaned assemblies are converted to lists of parental and child k-mers ($k = 3$ depicted). (**c**) Lists of k-mers are compared, and novel k-mers (the child's disjoint) are extracted and subjected to additional filtering. *Reference-based pipeline:* (**d**) Reads are aligned to a reference sequence and variants are called using maximum sensitivity settings and no application of filters. (**e**) Windows around putative variants are chosen. (**f**) Variants within window are combinatorically used generate all possible haplotypes, then distilled to k-mers of the same size used in the reference-free pipeline. No further filtering is applied. *Comparison:* (**g**) All k-mers from other sites in the genome are aggregated. The Venn diagram of k-mers from the reference-free and reference-based analyses is computed. (**h**) Shared k-mers reflect those that are present in the reference-free analysis and recapitulated by the reference-based analysis. Those unique to the reference-free analysis reflect those that could not be recapitulated by the reference-based analysis. The k-mers unique to the reference-based analysis are discarded.

variants collected. Assuming all sites are biallelic, this effectively imposed a theoretical limit of $2^{10} = 1024$ haplotypes from any given site. Additionally, as Delly emits a consensus haplotype sequence for structural variants precisely identified, we included this sequence in the list of haplotypes to be processed. We then emitted k-mers from these haplotypes, and advanced to the next unprocessed variant. These steps are depicted in Figure S1d-f.

In our *P. falciparum* data, the restriction of processing the first 10 variants seen within each 50-bp window affected $0.26\% \pm 0.06\%$ of windows processed per sample (23277 ± 4325 windows per sample, 62 ± 19 windows reduced per sample).

S1.3 Comparison

Finally, we examined the Venn diagram of reference-free and reference-based novel k-mer lists, identifying shared k-mers (present in both analyses) and reference-free-unique k-mers (novel k-mers not recapitulated by the reference-based analysis). Novel k-mers unique to the reference-based analysis were discarded as these largely originate from the combinatoric haplotype construction procedure and were not true genomic sequences. Figure S1g-h show these final comparison steps.

S2 Methods for DNM discovery based on linked genome assembly graphs

S2.1 Overview

Our DNM calling strategy is based on identifying mutational motifs in a "multi-color linked de Bruijn graph", or LdBG(9; 26). This can be decomposed into three steps. First, we construct LdBGs from short-read and long-haplotype datasets. Second, for each so-called "novel" k-mer (those unique to a child and absent from its parents), we assemble a child contig and one or more parental contigs containing k-mers shared with the child contig. Finally, we perform probabilistic all-to-all alignment allowing for recombination, attempting to describe the child's sequence as a series of match, insertion, deletion, and recombination operations on a panel of candidate parental sequences. Decoding the traceback of the probabilistic alignment yields variant calls. Further detail on each step is provided below.

S2.2 Construction of the linked de Bruijn graph

Briefly, a de Bruijn graph for sample c is formulated as a set of vertices and edges, $\mathcal{G}_c = \{\mathcal{V}_c, \mathcal{E}_c\}$. Vertices $\mathcal{V}_c$ are input sequences are broken into fixed length substrings of length k ("k-mers") with unit stride, and edges $\mathcal{E}_c$ encode $k-1$ overlaps of adjacent vertices. Each record is recorded as three columns: a k-mer sequence, its coverage, and its incoming/outgoing edges. N sample graphs constructed at identical k can be "stacked" by performing a full (outer) join on k-mer sequences, each sample c's coverage and edge information simply being recorded as two additional columns in each k-mer record. Stacking facilitates easy comparison of the graphs of N samples and formally yields a union graph $\mathcal{G} = \bigcup_{c=1}^{N} \mathcal{G}_c$. This formulation encodes relationships between two adjacent k-mers (the i-th and $(i+1)$-th k-mers in a sequence, as well as the $(i-1)$-th and i-th), but relationships between non-adjacent k-mers are lost. Thus, even if an input sequence spans a repeat when a single k-mer does not, the connectivity information inherent in the sequence is not retained. We restore this connectivity by trivially aligning input sequences $\mathcal{R}_{c,d}$ from dataset d to graph $\mathcal{G}_c$. The addition of new vertices to the graph during alignment is disallowed; the process merely amounts to lookups of shared k-mers between the input sequence and the graph, and bridging gaps over sequence differences with simple walks on $\mathcal{G}_c$. For all junctions (vertices with in-degree or out-degree > 1) spanned by an input sequence, we record the series of disambiguating edge choices (referred to as "links"), exhaustively annotating all participating junctions with relevant navigation information. We refer to this composite data structure (graph and links) as a linked de Bruijn graph, $\mathcal{G}_c = \{\mathcal{V}_c, \mathcal{E}_c, \bigcup_{d=1}^{D} \mathcal{L}_{c,d}\}$, where $\mathcal{L}_{c,d}$ is a sparse set of links on graph color c derived from sequence dataset d.

S2.3 Using links during LdBG navigation

By exhaustively annotating all spanned junctions with links, we ensure that traversal initiated anywhere in the graph has access to complete link information. Upon initiating a walk at vertex v_c, we collect each link we encounter. At a junction, we consult our list and extract the oldest link (i.e. the link that was obtained earliest in the traversal), as this link establishes the greatest context as to location in the genome. If there are multiple links with the same age that disagree as to the next junction choice, we halt traversal.

S2.4 Identification and filtration of novel *k*-mers

In a multi-color de Bruijn graph representing parents and children from a pedigree or an experimental cross, the locations of most DNMs will be signalled by the presence of novel *k*-mers: sequences unique to a child's genome and absent from both parental genomes. The set of novel *k*-mers in a child should also provide an indication as to how much novelty in a genome remains to be explained by some mutational process. As sequencing errors and sample contamination will also contribute to the set of novel *k*-mers, we sought to identify all novel *k*-mers in a child's graph and remove potential errors and contaminants. We identified and developed filters for five common graph or sequence motifs indicative of error:

1. **Contamination**: Contamination presents as a subset of novel *k*-mers that are unique to the sequencing data for a child but are irrelevant to the study at hand. To remove these sequences, each entry in the initial set of putative novel *k*-mers was screened for contamination via BLAST(1). We rejected any *k*-mer with a match of any quality to an organism other than the species under study. To account for mutations present in our contaminants but absent in the BLAST database, we used the contaminating *k*-mers as starting points for depth-first searches (DFS) in our graphs, exploring the child's graph until it rejoins a parent's graph, and rejecting all *k*-mers along the way.
2. **Graph tips**: Graph tips present as a series of novel *k*-mers that bifurcate from a parental graph but never rejoin. They are typically the result of sequencing errors at the ends of reads, but could also reflect true variation and subsequent coverage drop-out during sequencing. However, in the latter case, such variation tagged by novel *k*-mers would still not be recoverable without further sequencing data to fill in the missing coverage. To remove graph tips, we perform DFS from a putative novel *k*-mer, expecting to rejoin a parental graph on both ends. If exploration on one end connects to a parent and fails on the other end, we reject all child *k*-mers contained in the traversal.
3. **Promiscuously connected sequences**: Low-complexity sequence (or "dust") may manifest as *k*-mers promiscuously connected to many other low-complexity *k*-mers, presenting as a unnavigable graphical tangle. We defined such dust *k*-mers as those having a sum of in-degrees and out-degrees greater than 4. We initiated DFS at such *k*-mers, exploring until we either run out of edges to navigate or rejoin a parental graph, and keeping track of the number of *k*-mers traversed since the last time we observed one of low-complexity. If we reach one of the aforementioned stopping conditions and the distance traversed since the last low-complexity *k*-mer is less than the graph's *k*-mer size, we consider the traversed vertices to be dust and reject all elements.
4. **Highly-compressible sequence**: Additional low-complexity sequences are detected by computing the compression ratio ("CR") of the *k*-mer (gzip-compressed length vs uncompressed length) and removing any putative novel *k*-mer with a CR less than a predefined threshold (by default 0.703 for 47-bp *k*-mers).
5. **Orphans**: Graphical orphans are a series of novel *k*-mers that fail to ever connect to a parental graph. They may include contaminants absent from the BLAST database or reads with unusually high sequencing error. We performed DFS at putative novel *k*-mers, rejecting *k*-mers from traversals that joined one of the parental colors at any time.

We also removed putative novel *k*-mers from consideration based on two additional criteria:

1. **Shared k-mers**: Putative novel k-mers, while absent from parents, may be shared amongst children. Some of these may reflect recurrent *de novo* mutations, but the overwhelming majority stem from recurrent sequencing errors. We remove k-mers shared with other children (omitting clones of a child from consideration).
2. **Low coverage**: A number of putative novel k-mers substantially less than the mean coverage of the sample. Such k-mers may still permit navigation to flanking regions with coordinates in a parental genome, despite arising from sequencing error. We remove k-mers with coverage less than a specified value (by default, $6x$).

The bulk of sequences captured by these final two filters are likely to be recurrent sequencing error. However, we note that they could also remove a small number of DNMs from our consideration.

S2.5 Query sequence assembly

To construct sequences spanning putative variants, we perform contig assembly at each novel k-mer on the query sample (e.g. the child). Unless otherwise specified, these assemblies are conducted using McCortex links generated by threading the sample's paired-end read data and the parental assembly data through the query sample's graph(27). Optionally during graph traversal, if we encounter a junction vertex that (1) is itself a novel k-mer, (2) cannot be traversed with links, (3) one (and only one) of the outgoing vertices is also a novel k-mer, then we assume both novel k-mers are part of the same mutational event and extend contig construction through these vertices. As assemblies seeded by proximate novel k-mers may result in redundant contigs, we post-process the contig set to remove redundant sequences and those fully contained by other contigs. Finally, if multiple contigs share a novel k-mer, we remove all but the contig that contains the largest number of novel k-mers. This effectively "partitions" the contig set into those representing distinct mutational events.

S2.6 Source sequence assembly

For each query sequence, we build a panel of source sequences to which the query is aligned. At each non-novel k-mer in the query sequence, we perform contig assembly on the source samples (e.g. the parents). Unless otherwise specified, these assemblies are conducted using McCortex links generated by threading the sample's paired-end read data and the parental assembly data through the child's graph. During assembly, gaps at the boundaries of mutational events in the query sample may be incompletely assembled due to sequencing error or graph homology. We close these gaps via DFS between gap boundaries. If still not closed, we assemble gap flanks by a maximum of 500 bp. Flanking sequence irrelevant to the query is trimmed by subsetting the source within the boundaries of the earliest and latest k-mers shared with the query sequence.

Each source sequence is given a unique label, simply incrementing from first to last. If a reference sequence is specified for the relevant sample in the LdBG, the source sequence is aligned to that reference using `bwa mem` and relabelled with the resulting genomic coordinates. Note that the relabelling step does not alter the source sequence in any way.

S2.7 Variant typing by simultaneous alignment to reference genome panels

Two general classes of graphical variant motifs concern us: "bubbles" (SNVs, short indels and inversions, multi-nucleotide polymorphisms), and "breakends" (large indels and inversions, non-

allelic homologous recombinations, gene conversions, and allelic recombinations). We address both classes of variants in a single probabilistic framework wherein a novel k-mer-spanning contig ("query" sequence) is simultaneously aligned to a panel of candidate haplotypes ("source" sequences). We achieve this by repurposing the Tesserae model (14, supplementary methods), a pair-HMM combining models for global alignment with affine gap penalty (described in 5) and haplotype diversity estimation via recombination(13), to the task of bubble and breakpoint variant typing.

The model (including formal descriptions of the Viterbi, Forward, and Backward algorithms) is fully specified in Zilversmit *et al.* 2013. Briefly, we assume a query sequence arises as an imperfect mosaic of source sequences. For each query and its candidate source sequences (collectively referred to as the "sequence set", h), we apply the Viterbi algorithm to find the maximum likelihood path through our pair-HMM. The pair-HMM is specified by a transition matrix and emission matrix, provided below. Terms are defined as follows:

Table S1. Definitions and defaults in the Tesserae pair-HMM.

Term	Definition	Default
δ	probability of indel initiation	0.025
ϵ	probability of indel extension	0.75
ρ	probability of recombination	0.0001
π_M	probability of starting in match (M) state	0.75
π_I	probability of starting in insert (I) state	$1 - \pi_M$
τ	probability of termination	0.001
$\|Y\|$	$\Sigma_{k=1}^{n} l_k$ where l_k is the length of sequence k	-

The transition matrix is given by:

	B	M_x	I_x	D_x	M_k	I_k	D_k	T
B	0	$\frac{\pi_M}{\|Y\|}$	$\frac{\pi_I}{\|Y\|}$	0	$\frac{\pi_M}{\|Y\|}$	$\frac{\pi_I}{\|Y\|}$	0	0
M_x	0	$1-2\delta-\rho-\tau$	δ	δ	$\frac{\rho\pi_M}{\|Y\|}$	$\frac{\rho\pi_I}{\|Y\|}$	0	τ
I_x	0	$1-\epsilon-\rho-\tau$	ϵ	0	$\frac{\rho\pi_M}{\|Y\|}$	$\frac{\rho\pi_I}{\|Y\|}$	0	τ
D_x	0	$1-\epsilon$	0	ϵ	0	0	0	0
M_k	0	$\frac{\rho\pi_M}{\|Y\|}$	$\frac{\rho\pi_I}{\|Y\|}$	0	$1-2\delta-\rho-\tau$	δ	δ	τ
I_k	0	$\frac{\rho\pi_M}{\|Y\|}$	$\frac{\rho\pi_I}{\|Y\|}$	0	$1-\epsilon-\rho-\tau$	ϵ	0	τ
D_k	0	0	0	0	$1-\epsilon$	0	ϵ	0
T	0	0	0	0	0	0	0	1

The emission probability of a nucleotide emitted from an insertion state is $e(x_i) = 0.2$, while the nucleotide emission matrix $e(x_i, y_j)$ (where x_i is the nucleotide at site i in sequence x and y_j is the nucleotide at site j in sequence y) is given by:

$$e(x_i, y_j) = \begin{cases} 0.9 & x_i = y_j \\ 0.05 & \text{if nucleotide transition} \\ 0.025 & \text{if nucleotide transversion} \end{cases} .$$

The trellis diagram for the model is provided in Figure S2.

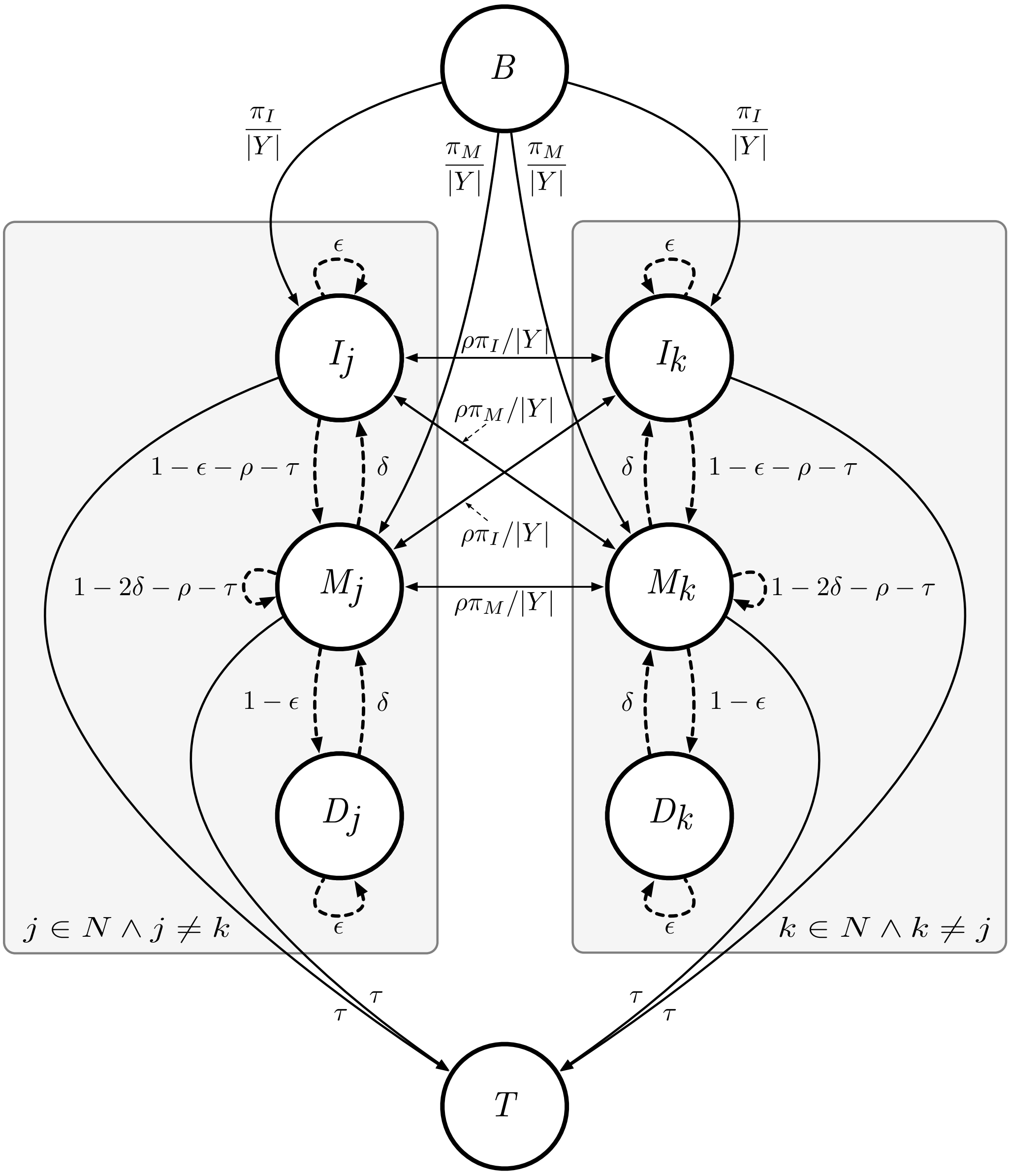

Figure S2. The Tesserae model trellis diagram for simultaneous alignment with recombination. Each plate denotes a global alignment model for input query and source sequences. Match, insert, and delete states are connected within single sequences. To permit recombination, all sequences are exhaustively connected through match and insert (but not delete) states. Dashed lines indicate internal (intra-plate) transitions. Solid lines indicate external (inter-plate) transitions.

By keeping pointers through the recursion process of the Viterbi algorithm, we trace back the most probable path through h. This path through the resulting traceback matrix can be interpreted as specifying the background haplotype for subsequences and variants in the query sequence against this background. Figure S3 depicts the overall process on a toy example consisting of a short query sequence and two candidate source sequences. The query sequence is constructed by initiating assembly on the LdBG in the query color at one or more novel k-mers. The two candidate source haplotypes are constructed by initiating assembly at non-novel k-mers

found in the query sequence. Redundant contigs are removed (this step is not shown). The sequence set is processed with the Tesserae model, and decoding of the resulting traceback matrix reveals two recombination breakends and an insertion.

```
a.          query   CGAACAGGATGTAGGCGAGATGACGCCATTTATTCTTTTCGTGCATAACAAAACGATAGTAG
          source0   CGAACAGGATCAGGGATAAAACAAATTGATTATTCTTTTCGTGCATAACACGATAGTAG
          source1 GTCATACGACCGTAGGCGAGATGACGCCATTTATTACGGATATTATATTTATATA

b.   query   (0-61) CGAACAGGATGTAGGCGAGATGACGCCATTTATTCTTTTCGTGCATAACAAAACGATAGTAG
                    ||||||||||||||||||||||||||||||||||||||||||||||||^^^||||||||||
   source0    (0-9) CGAACAGGAT
   source1 (11-34)            GTAGGCGAGATGACGCCATTTATT
   source0 (34-58)                                    CTTTTCGTGCATAAC---ACGATAGTAG

                              ↓                        ↓                 ↓
c.                           BND                      BND               INS
```

Figure S3. Assembly, alignment, and variant decoding process for a toy example. (**a**) A 61-bp query sequence with three regions of sequence novelty (nucleotides with a red background). To assemble candidate source sequences, k-mers in the non-novel regions are used to seed contig traversals on the LdBG in the source colors (in this example, $k = 7$). Sequences with yellow backgrounds indicate the seed k-mers for these traversals. For visual clarity, sequences shown here are positioned manually, and source subsequences that are not present in the query sequence are shown in grey. (**b**) All sequences in the sequence set aligned with the Tesserae model, with breakends depicted as linebreaks and variants shown on their haplotypic background. (**c**) Putative mutations spanned by novel k-mers are emitted as breakend ("BND") variant calls.

S3 Data processing: long-read sequencing, assembly, and alignment

S3.1 Data availability

PacBio RSII raw read ERA accession numbers, processed assembly download paths, and annotation file download paths are provided below:

Table S2. Pacbio read and assembly metadata and download paths.

source code	secondary sample accession	origin	culturing	sequencing	chemistry	fasta
HB3	ERS712858	Honduras	SI	SI	P6C4	FASTA
DD2	ERS639545	Vietnam	SI	SI	P6C4	FASTA
7G8	ERS686280	Brazil	SI	SI	P6C4	FASTA
GB4	ERS3566948	Ghana	NIH	CSHL	P6C4	FASTA
803	ERS3566949	Cambodia	NIH	CSHL	P6C4	FASTA
36F11	ERS3119776	803xGB4 progeny	NIH	CSHL	P6C4	FASTA

A machine-readable version of this manifest is available at `https://github.com/mcveanlab/CortexJDK/blob/master/manuscript/manifest.pacbio.txt`.

S3.2 Library preparation

To facilitate draft reference genome construction, we obtained high molecular weight genomic DNA (HMW gDNA) for seven *P. falciparum* parasites: all six parental clones spanning the four experimental crosses (3D7, HB3, DD2, 7G8, GB4, 803) and one progeny clone from the 803xGB4 cross (36F11). Parasite lines for 3D7, HB3, DD2, and 7G8 were produced by the Kwiatkowski lab at the Wellcome Trust Sanger Institute (SI). Lines for 803, GB4, and 36F11 were produced by the National Institute of Allergy and Infectious Diseases at the NIH (NIH). All cultures were maintained under standard conditions(25).

We obtained $6-20$ μg of HMW gDNA from each haploid parasite culture. QC was performed with NanoDrop spectrophotometers (Thermo Fisher Scientific) to verify gDNA purity. 20 kbp insert SMRTbell libraries were generated per sample with a Blue-Pippin (Sage Science) size selection range of $10-50$ kbp.

For additional assembly polishing of the HB3, DD2, and 7G8 genomes, we generated short fragment libraries using 0.5 μg of DNA and a PCR-free library construction method(10). These libraries were sequenced using on MiSeq Illumina instruments, generating 250 bp paired-end reads and mean fragment length of 500 bp.

PacBio RSII sequencing of each library took place at two facilities. 3D7, GB4, 803, and 36F11 were sequenced at Cold Spring Harbor Laboratory's Next Generation Genomics Shared Resource (CSHL). HB3, DD2, and 7G8 were sequenced at the Sanger Institute.

S3.3 *De novo* assembly

3D7, HB3, DD2, and 7G8 assemblies on the libraries described above have been previously reported and publicly released(17). Summarizing, raw reads were assembled using the HGAP2 software using default parameters and a genome size of 23.5 Mbp. The HGAP assemblies were

further improved by removing contaminating sequences, short contigs, and overlapping contigs; merging contigs with substantial overlap length; and polishing using the 250 bp Illumina reads aligned to the assembly.

Remaining samples GB4, 803, and 36F11 were assembled with the HGAP3 software.

S3.4 Contaminant removal

To remove possible contaminants from all assemblies, we ran contigs through BLAST(2), excluding any contig with a match to an organism other than *P. falciparum* in the `nt` database (updated Oct 2017).

S3.5 Pseudochromosome contiguation and gene annotation

To reorient contigs to match canonical reference orientation, build scaffolds representing whole chromosomes, and annotate genes, we ran all assemblies through the Companion webserver(24). We set the `Reference organism` setting as `Plasmodium falciparum 3D7`. We specified that both *ab initio* gene finding and existing gene model transfer be performed, specifying the `Strain` option for the RATT(18) gene model transfer tool. All other settings remained as software defaults. Note that Companion produced scaffolds representing all 14 autosomes in the *P. falciparum* genome, but does not automatically recognize and circularize the mitochondria and apicoplast genomes. These linear contigs, along with contigs that could not be placed in an autosome, are concatenated in a separate contig named with a "_00" suffix. Hence, scaffolding by pseudochromosome contiguation results in 15 total scaffolds per assembly.

S3.6 Annotation of repetitive regions and accessory compartments

We annotated repetitive sequences by applying the RepeatMasker(23) software to each genome using the maximum sensitivity `-s` option and the `-species 'plasmodium falciparum'` argument. We annotated core and accessory regions of each genome with the (19) software, processing each chromosome across all parental genomes simultaneously.

S3.7 Quality assessment

We first estimated the overall quality of our assemblies by performing a scaffold-to-chromosome alignment of our proof-of-principle 3D7 draft to the finished reference genome of the same parasite(6) using MUMmer(11). The alignments are visualized as a multi-dotplot in Figure S4, an extension of a dot plot that depicts alignments as two dimensional matrices with target and query sequences on the x and y axes respectively(7). Most chromosomes are assembled completely, and the overwhelming majority of the assembly appears on-diagonal (indicating successful one-to-one reconstruction). Elements appearing off-diagonal could represent misassembly. However, note that most of these off-diagonal elements occur towards the extremes of each chromosome. Given that the reference genome was constructed with Sanger reads an order of magnitude shorter than the PacBio reads, it is possible some repetitive regions have been collapsed or misplaced, contributing to this nominal error rate.

We called variants between the two assemblies to quantify errors using MUMmer, finding $3,357$ SNPs, $11,620$ insertions, and $4,603$ deletions. Overall, the SNP, insertion, and deletion rates are exceedingly low: amounting to $19,580$ events in a 23 Mbp genome (0.17%). The insertion

rate is much higher than that of deletions and SNPs, perhaps due to the dominant insertion error mode of the PacBio sequencing instrument. All chromosomes appear reasonably similar in performance. Based on these measurements of the error rate, we estimate the quality of the PacBio assembly of the 3D7 isolate to be approximately Q31[1], or less than one error per thousand bases.

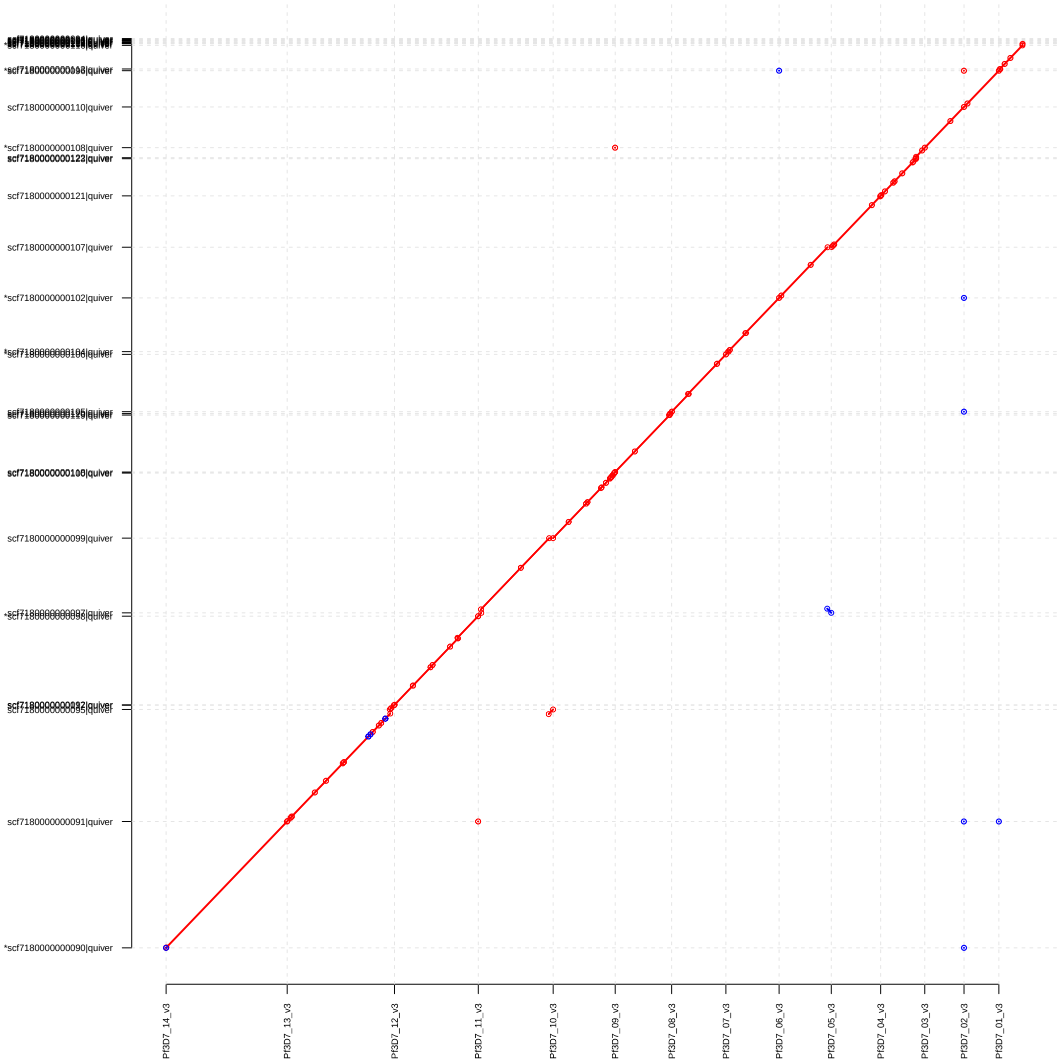

Figure S4. Alignment of contigs from the 3D7 draft to the finished reference assembly, with sequences from reference and draft assembly on the x and y-axis, respectively. Each contig from the draft assembly is represented by a line segment terminated at either end with circles. Red denotes forward alignment of contigs to the reference, blue denotes reverse alignment.

As we did not have high-quality, finished genomes against which to compare the other assemblies, we devised a quality metric approximation based on k-mer counting. We used our graph processing software to find k-mers present in the PacBio assembly and absent in the Illumina

[1] $Q = -10\log_{10}(q) = -10\log_{10}((11,620 + 4,603 + 3,357)/23,332,831)$

Table S3. Metrics on PacBio sequencing and assemblies

	3D7 (ref)	3D7 (draft)	HB3	DD2	7G8	GB4	803	36F11
Assembler	–	HGAP2	HGAP2	HGAP2	HGAP2	HGAP3	HGAP3	HGAP3
Contigs	–	34	28	16	17	104	35	33
Scaffolds[a]	16	15	15	15	15	15	15	15
Length (bp)	23332839	23729641	22813863	22682439	22832395	27105702	23074973	23429077
Length (autosomes) (bp)	22671410	23194002	22671410	22641998	22775193	24613844	22772320	22989675
Core genome (bp)	20574502	–	21047328	21058329	21137184	21514228	21093156	–
Accessory genome (bp)	2096908	–	1624082	1583669	1638009	3099616	1679164	–
Genes (autosomes)	5561	5141	5130	5166	5219	5041	4501	4174
Quality (MUMmer)	–	31	–	–	–	–	–	–
Quality (Graph)	–	28	29	31	27	23	28	25

[a] In the draft reference sequences, the circular mitochondrial and apicoplast genomes are grouped together in a non-autosomal scaffold, resulting in one less scaffold than the canonical reference.

data for the same sample. We retained only one *k*-mer per variant event, discarding adjacent *k*-mers that tag the same event. We computed assembly quality using the number of retained *k*-mers as an estimate of the number of bases different between the PacBio and Illumina data.

We note that both measurements of assembly quality are pessimistic estimates. Our MUMmer-based estimates are predicated on the assumption that any differences between our 3D7 assembly and the canonical reference assembly indicate errors in our draft. In long, repetitive regions of the genome, this assumption may not be accurate as the Sanger reads used to assemble the reference were an order of magnitude shorter than the PacBio reads, potentially leading to collapsed repeats. Similarly for the *k*-mer counting estimate, while the PacBio RSII reads are known to have a much higher error rate than Illumina reads, it is not necessarily the case that all discrepancies between PacBio assemblies and Illumina reads should be adjudicated in favor of the latter.

Per-sample sequencing and final assembly details are presented in Table S3.

S4 Data processing: short-read sequencing, alignment, and assembly

Table S4: Illumina metadata and download paths.

cross	sample	source code	accession	fastq end1 ftp	fastq end2 ftp
3D7xHB3	PG0051-C	3D7_Glasgow	ERR019061	FASTQ (END1)	FASTQ (END2)
3D7xHB3	PG0052-C	HB3_Glasgow	ERR019054	FASTQ (END1)	FASTQ (END2)
3D7xHB3	PG0053-C	XP3	ERR019067	FASTQ (END1)	FASTQ (END2)
3D7xHB3	PG0054-C	XP4	ERR019062	FASTQ (END1)	FASTQ (END2)
3D7xHB3	PG0055-C	XP5	ERR019066	FASTQ (END1)	FASTQ (END2)
3D7xHB3	PG0056-C	XP8	ERR019068	FASTQ (END1)	FASTQ (END2)
3D7xHB3	PG0057-C	XP9	ERR019069	FASTQ (END1)	FASTQ (END2)
3D7xHB3	PG0058-C	XP24	ERR019063	FASTQ (END1)	FASTQ (END2)
3D7xHB3	PG0060-C	XP52	ERR019058	FASTQ (END1)	FASTQ (END2)
3D7xHB3	PG0061-C	X2	ERR019059	FASTQ (END1)	FASTQ (END2)
3D7xHB3	PG0062-C	X4	ERR019070	FASTQ (END1)	FASTQ (END2)
3D7xHB3	PG0063-C	X5	ERR019060	FASTQ (END1)	FASTQ (END2)
3D7xHB3	PG0064-C	X6	ERR019071	FASTQ (END1)	FASTQ (END2)
3D7xHB3	PG0065-C	X10	ERR019064	FASTQ (END1)	FASTQ (END2)
3D7xHB3	PG0066-C	X11	ERR019072	FASTQ (END1)	FASTQ (END2)
3D7xHB3	PG0067-C	X12	ERR019073	FASTQ (END1)	FASTQ (END2)
3D7xHB3	PG0068-C	X30	ERR019065	FASTQ (END1)	FASTQ (END2)
3D7xHB3	PG0069-C	X33	ERR019055	FASTQ (END1)	FASTQ (END2)
3D7xHB3	PG0070-C	X35	ERR019056	FASTQ (END1)	FASTQ (END2)
3D7xHB3	PG0071-C	X39	ERR019074	FASTQ (END1)	FASTQ (END2)
HB3xDD2	PG0004-CW	HB3_Ferdig	ERR012788	FASTQ (END1)	FASTQ (END2)
HB3xDD2	PG0008-CW	DD2_Ferdig	ERR012840	FASTQ (END1)	FASTQ (END2)
HB3xDD2	PG0015-C	B1SD	ERR019044	FASTQ (END1)	FASTQ (END2)
HB3xDD2	PG0016-C	QC13	ERR012895	FASTQ (END1)	FASTQ (END2)
HB3xDD2	PG0017-C	QC01	ERR019050	FASTQ (END1)	FASTQ (END2)
HB3xDD2	PG0018-C	B4R3	ERR019042	FASTQ (END1)	FASTQ (END2)
HB3xDD2	PG0019-C	SC05	ERR019051	FASTQ (END1)	FASTQ (END2)
HB3xDD2	PG0020-C	TC08	ERR019052	FASTQ (END1)	FASTQ (END2)
HB3xDD2	PG0021-C	GC03	ERR015447	FASTQ (END1)	FASTQ (END2)
HB3xDD2	PG0024-C	3BD5	ERR019053	FASTQ (END1)	FASTQ (END2)
HB3xDD2	PG0027-C	TC05	ERR015450	FASTQ (END1)	FASTQ (END2)
HB3xDD2	PG0030-C	7C188	ERR019046	FASTQ (END1)	FASTQ (END2)
HB3xDD2	PG0031-C	7C408	ERR015458	FASTQ (END1)	FASTQ (END2)
HB3xDD2	PG0034-C	7C3	ERR019047	FASTQ (END1)	FASTQ (END2)
HB3xDD2	PG0037-C	7C20	ERR015451	FASTQ (END1)	FASTQ (END2)
HB3xDD2	PG0038-C	7C111	ERR015457	FASTQ (END1)	FASTQ (END2)
HB3xDD2	PG0039-C	7C140	ERR015454	FASTQ (END1)	FASTQ (END2)
HB3xDD2	PG0040-Cx	7C159	ERR107475	FASTQ (END1)	FASTQ (END2)
HB3xDD2	PG0041-C	7C170	ERR015446	FASTQ (END1)	FASTQ (END2)

HB3xDD2	PG0042-C	7C183	ERR015448	FASTQ (END1)	FASTQ (END2)
HB3xDD2	PG0043-C	7C421	ERR015459	FASTQ (END1)	FASTQ (END2)
HB3xDD2	PG0044-C	7C424	ERR019043	FASTQ (END1)	FASTQ (END2)
HB3xDD2	PG0045-C	QC23	ERR012892	FASTQ (END1)	FASTQ (END2)
HB3xDD2	PG0046-Cx	7C46	ERR107476	FASTQ (END1)	FASTQ (END2)
HB3xDD2	PG0047-C	7C126	ERR015452	FASTQ (END1)	FASTQ (END2)
HB3xDD2	PG0048-C	7C7	ERR019049	FASTQ (END1)	FASTQ (END2)
7G8xGB4	PG0077-C	JC3	ERR027112	FASTQ (END1)	FASTQ (END2)
7G8xGB4	PG0077-CW	JC3	ERR045636	FASTQ (END1)	FASTQ (END2)
7G8xGB4	PG0078-C	QF5	ERR029092	FASTQ (END1)	FASTQ (END2)
7G8xGB4	PG0078-CW	QF5	ERR045638	FASTQ (END1)	FASTQ (END2)
7G8xGB4	PG0079-C	JF6	ERR027102	FASTQ (END1)	FASTQ (END2)
7G8xGB4	PG0079-CW	JF6	ERR045637	FASTQ (END1)	FASTQ (END2)
7G8xGB4	PG0080-C	TF1	ERR027103	FASTQ (END1)	FASTQ (END2)
7G8xGB4	PG0081-CW	DEV_18_05_11	ERR045633	FASTQ (END1)	FASTQ (END2)
7G8xGB4	PG0082-C	WC4	ERR029093	FASTQ (END1)	FASTQ (END2)
7G8xGB4	PG0083-C	7G8_NIH	ERR027099	FASTQ (END1)	FASTQ (END2)
7G8xGB4	PG0084-C	GB4_NIH	ERR027100	FASTQ (END1)	FASTQ (END2)
7G8xGB4	PG0087-C	JB8	ERR029091	FASTQ (END1)	FASTQ (END2)
7G8xGB4	PG0088-C	KH7	ERR027111	FASTQ (END1)	FASTQ (END2)
7G8xGB4	PG0090-C	KC2	ERR027116	FASTQ (END1)	FASTQ (END2)
7G8xGB4	PG0091-C	KA6	ERR027117	FASTQ (END1)	FASTQ (END2)
7G8xGB4	PG0093-C	XB3	ERR029105	FASTQ (END1)	FASTQ (END2)
7G8xGB4	PG0094-C	D2	ERR027106	FASTQ (END1)	FASTQ (END2)
7G8xGB4	PG0094-CW	D2_18_05_11	ERR045632	FASTQ (END1)	FASTQ (END2)
7G8xGB4	PG0095-C	NIC	ERR027107	FASTQ (END1)	FASTQ (END2)
7G8xGB4	PG0096-C	NF10	ERR027108	FASTQ (END1)	FASTQ (END2)
7G8xGB4	PG0097-C	WF12	ERR027109	FASTQ (END1)	FASTQ (END2)
7G8xGB4	PG0098-C	DAN	ERR027110	FASTQ (END1)	FASTQ (END2)
7G8xGB4	PG0099-C	JB12	ERR029146	FASTQ (END1)	FASTQ (END2)
7G8xGB4	PG0100-C	JE11	ERR029404	FASTQ (END1)	FASTQ (END2)
7G8xGB4	PG0101-C	KC5	ERR029147	FASTQ (END1)	FASTQ (END2)
7G8xGB4	PG0102-C	XF12	ERR029143	FASTQ (END1)	FASTQ (END2)
7G8xGB4	PG0102-CW	XF12_18_05_11	ERR045635	FASTQ (END1)	FASTQ (END2)
7G8xGB4	PG0104-C	KB8	ERR029148	FASTQ (END1)	FASTQ (END2)
7G8xGB4	PG0105-C	XD8	ERR029144	FASTQ (END1)	FASTQ (END2)
7G8xGB4	PG0105-CW	XD8_13_05_11	ERR045628	FASTQ (END1)	FASTQ (END2)
7G8xGB4	PG0107-C	JON	ERR029408	FASTQ (END1)	FASTQ (END2)
7G8xGB4	PG0109-C	XG10	ERR029405	FASTQ (END1)	FASTQ (END2)
7G8xGB4	PG0110-C	LC12	ERR171454	FASTQ (END1)	FASTQ (END2)
7G8xGB4	PG0111-C	JC9	ERR029409	FASTQ (END1)	FASTQ (END2)
7G8xGB4	PG0111-CW	JC9_18_05_11	ERR045634	FASTQ (END1)	FASTQ (END2)
7G8xGB4	PG0112-C	AUD	ERR029406	FASTQ (END1)	FASTQ (END2)
7G8xGB4	PG0113-CW	JH6_12_05_11	ERR045626	FASTQ (END1)	FASTQ (END2)

803xGB4	PG0443-C	803	ERR570006 FASTQ (END1) FASTQ (END2)
803xGB4	PG0050-CX2	GB4	ERR570014 FASTQ (END1) FASTQ (END2)
803xGB4	PG0445-C	11H5	ERR570030 FASTQ (END1) FASTQ (END2)
803xGB4	PG0446-C	36F11	ERR570038 FASTQ (END1) FASTQ (END2)
803xGB4	PG0447-C	36H9	ERR656280 FASTQ (END1) FASTQ (END2)
803xGB4	PG0448-C	37D9	ERR656283 FASTQ (END1) FASTQ (END2)
803xGB4	PG0450-C	39A4	ERR656289 FASTQ (END1) FASTQ (END2)
803xGB4	PG0451-C	39C5	ERR656292 FASTQ (END1) FASTQ (END2)
803xGB4	PG0453-C	43H3	ERR656298 FASTQ (END1) FASTQ (END2)
803xGB4	PG0455-C	46G9	ERR570015 FASTQ (END1) FASTQ (END2)
803xGB4	PG0458-C	61A12	ERR570039 FASTQ (END1) FASTQ (END2)
803xGB4	PG0459-C	61D3	ERR656281 FASTQ (END1) FASTQ (END2)
803xGB4	PG0460-C	61E6	ERR656284 FASTQ (END1) FASTQ (END2)
803xGB4	PG0461-C	61E8	ERR656287 FASTQ (END1) FASTQ (END2)
803xGB4	PG0462-C	71D6	ERR656290 FASTQ (END1) FASTQ (END2)
803xGB4	PG0463-C	76H10	ERR656293 FASTQ (END1) FASTQ (END2)
803xGB4	PG0464-C	85D3	ERR656296 FASTQ (END1) FASTQ (END2)
803xGB4	PG0465-C	87A11	ERR656299 FASTQ (END1) FASTQ (END2)
803xGB4	PG0466-C	87E7	ERR570008 FASTQ (END1) FASTQ (END2)
803xGB4	PG0467-C	40G11	ERR570016 FASTQ (END1) FASTQ (END2)
803xGB4	PG0469-C	50C5	ERR570032 FASTQ (END1) FASTQ (END2)
803xGB4	PG0470-C	24G11	ERR570040 FASTQ (END1) FASTQ (END2)
803xGB4	PG0471-C	36D5	ERR656282 FASTQ (END1) FASTQ (END2)
803xGB4	PG0472-C	36E5	ERR656285 FASTQ (END1) FASTQ (END2)
803xGB4	PG0473-C	38G5	ERR656288 FASTQ (END1) FASTQ (END2)
803xGB4	PG0474-C	39C3	ERR656291 FASTQ (END1) FASTQ (END2)
803xGB4	PG0475-C	4E8	ERR656294 FASTQ (END1) FASTQ (END2)
803xGB4	PG0476-C	34F5	ERR656297 FASTQ (END1) FASTQ (END2)
803xGB4	PG0492-C	34B1	ERR905451 FASTQ (END1) FASTQ (END2)
803xGB4	PG0494-C	35C2	ERR905453 FASTQ (END1) FASTQ (END2)
803xGB4	PG0495-C	38A6	ERR905454 FASTQ (END1) FASTQ (END2)
803xGB4	PG0496-C	38E11	ERR905455 FASTQ (END1) FASTQ (END2)
803xGB4	PG0498-C	39G5	ERR905457 FASTQ (END1) FASTQ (END2)
803xGB4	PG0499-C	39H5	ERR905458 FASTQ (END1) FASTQ (END2)
803xGB4	PG0500-C	40A6	ERR905459 FASTQ (END1) FASTQ (END2)
803xGB4	PG0501-C	40B12	ERR905460 FASTQ (END1) FASTQ (END2)
803xGB4	PG0502-C	40F4	ERR905461 FASTQ (END1) FASTQ (END2)
803xGB4	PG0503-C	40G2	ERR905462 FASTQ (END1) FASTQ (END2)
803xGB4	PG0504-C	43E5	ERR905463 FASTQ (END1) FASTQ (END2)
803xGB4	PG0505-C	44D4	ERR905464 FASTQ (END1) FASTQ (END2)
803xGB4	PG0508-C	76H10-Tk13	ERR905468 FASTQ (END1) FASTQ (END2)
803xGB4	PG0509-C	85G7	ERR905469 FASTQ (END1) FASTQ (END2)
803xGB4	PG0510-C	88C9	ERR905470 FASTQ (END1) FASTQ (END2)
803xGB4	PG0512-C	37F12	ERR905473 FASTQ (END1) FASTQ (END2)

S4.1 Data availability

Raw reads for *P. falciparum* parents and progeny were provided to us by the MalariaGen project(16). ERA accession numbers and fastq download paths are provided in Table S4.

A machine-readable version of this manifest is available at `https://github.com/mcveanlab/CortexJDK/blob/master/manuscript/manifest.illumina.txt`.

S4.2 Library preparation

We obtained short-read, paired-end whole genome sequence data for parent and progeny clones of the 3D7xHB3(28), HB3xDD2(29), 7G8xGB4(8), and 803xGB4(22) crosses. All samples were sequenced on Illumina platforms between 2010 and 2014 using a PCR-free library preparation protocol intended to reduce coverage biases associated with AT-rich templates. Avoiding PCR during library construction also removes the issue of replication errors that occur in early cycles being propagated to all subsequent copies, thus masquerading as DNMs. Across all four crosses, and excluding parasite clones, we obtained 127 samples (8 parents, 119 progeny). The data is summarized in Table S5.

S4.3 Alignment

Table S5. Summary of sequencing data for four *P. falciparum* crosses

	3D7xHB3	HB3xDD2	7G8xGB4	803xGB4
Parents	2	2	2	2
Progeny	18	24	35	42
Read length (bp)	76	76	76	100
Insert size (bp)	300 ± 29	253 ± 48	293 ± 21	222 ± 10
Coverage	99 ± 38	121 ± 91	110 ± 40	205 ± 106
Platform	Illumina GAII	Illumina GAII	Illumina GAII	Illumina HiSeq 2000
Sequencing date	2010	2009-2012	2010-2011	2014

We obtained the *P. falciparum* reference sequence(6) obtained from PlasmoDB(3) (build 32.0, April 2017). For reference-based analyses, we aligned all reads with `bwa mem` to this reference using default settings. We marked duplicate reads using the `MarkDuplicates` tool in the Picard suite, and applied the GATK base quality score recalibrator (BQSR) to improve the accuracy of the base quality scores(4). We computed coverage metrics with the GATK's `DepthOfCoverage` tool, and insert size metrics with Picard's `CollectInsertSizeMetrics` tool[2]. A summary of the sample data is presented in Table S5.

S4.4 Reference-based variant calls

For later comparison to our de Bruijn graph-based DNM calls, we applied two software packages to the 20 samples in the 3D7xHB3 cross. For SNVs and small indels, we applied the GATK's

[2] `http://broadinstitute.github.io/picard/`

HaplotypeCaller(20), specifying a ploidy of 1 and leaving all other parameters as software defaults. For larger and more complex structural variants (insertions, deletions, tandem duplications, inversions, and translocations), we applied the Delly2 software(21). No additional filtering was applied to these callsets in order to preserve maximum sensitivity for later comparison to our *k*-mer-counting analyses.

S4.5 *De novo* assembly

We constructed multi-color linked de Bruijn graphs (LdBGs) for all 127 samples using McCortex(26) with a four-step pipeline. First, to build raw (un-error-cleaned) graphs, we applied the `build` command with a *k*-mer size parameter of 47 bp for all samples. Next, we ran the `clean` command with default settings to produce error-cleaned graphs. Substantial coverage variation in the *P. falciparum* samples caused the default error-cleaning process to erroneously remove true genomic *k*-mers. Unaddressed, this lead to gapped assemblies with unusually short contigs, as *k*-mers necessary for haplotype threading were not present in the graph. We implemented a repair module (`RecoverExcludedKmers`) in Corticall, recovering *k*-mers that were present in the raw graph, absent in the clean graph, and present in the canonical reference or either parental PacBio draft assembly for the cross. We ran the McCortex `inferedges` tool on the error-recovered graph, ensuring adjacencies between records in the graph were properly recorded. Finally, the parents, children, PacBio draft references, and reference genome were joined into a single multi-color graph to facilitate downstream processing using the Corticall `Join` command (a low-memory equivalent to McCortex's `join` command). All graphs were output in sorted order to facilitate random access over the dataset in Corticall.

We constructed three link annotation sets for each sample graph using the McCortex `thread` command: one for each parental PacBio draft and one for a sample's paired end reads. For PacBio assemblies and reads, we constructed links using the two-way gap-filling option. For paired-end reads, we further improved these links by re-threading links using `thread`'s paired-end mode and minimum (maximum) fragment size parameters 0 bp (1000 bp). We then filtered out low-coverage links likely arising from sequencing errors according to McCortex's documentation.

S5 Simulation of progeny genomes

We simulated progeny genomes from a synthetic crossing of HB3 and DD2 parasites with known allelic recombinations and DNMs using the Corticall module `SimulateHaploidChild`. Our simulations output a VCF describing recombination operations and other permutations to the appropriate reference sequences. The newly generated progeny reference genomes were then used to simulate reads for *de novo* assembly and DNM calling.

S5.1 Homologous recombination

We began with the pseudochromosome contiguated assemblies of HB3 and DD2 from section S3.5, for which homologous chromosomes suitable for recombination were easily paired. Crossover number c was taken to be Poisson-distributed, parameterized with values as determined in Miles *et al.* 2016(15) and shown in Equation 1 with rate parameter $\mu = \lambda d$, average crossover rate $\lambda = 0.0135\ cM^{-1}$ and map length $d(\ell) = 0.74\ell - 0.11$, where ℓ is chromosome length. Map length and crossover probability distributions are shown in Figure S5.

$$P\{c|\ell\} = \frac{\mu^c}{c!}e^{-\mu} = \frac{(\lambda d(\ell))^c}{c!}e^{-\lambda d(\ell)} \tag{1}$$

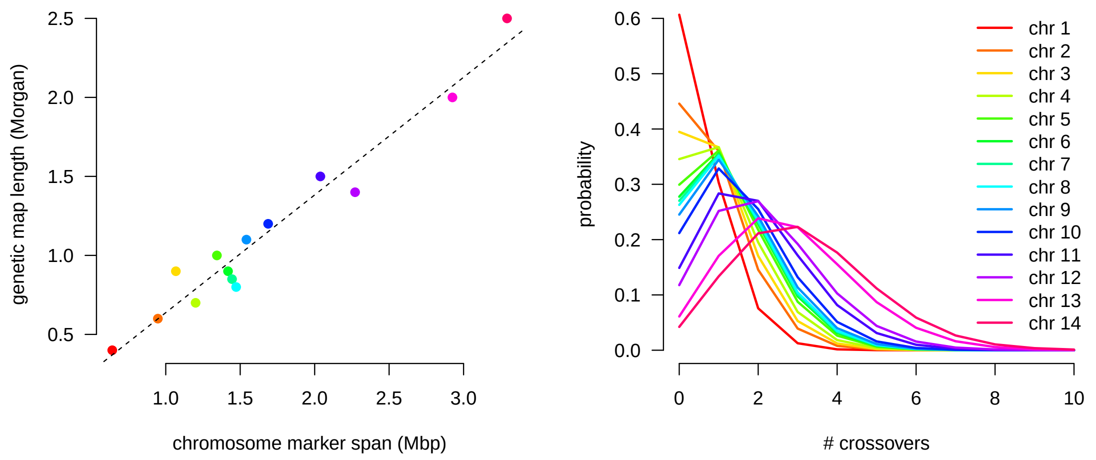

Figure S5. Simulated map lengths per chromosome (left) and crossover probability distribution per chromosome (right).

S5.2 Non-homologous allelic recombination

We simulated non-homologous allelic recombination (NAHR) events between members of the *var* gene family annotated in the HB3 and DD2 draft genome assemblies. While true NAHR events appear to be mediated by microhomology and possibly 5′ upstream promoter sequence, we deemed random pairings sufficient for our needs. For each simulated progeny, we chose at random two *var* genes from each genome, computed exact homology maps at $k = 21$ between the two sequences, and simulated a random number (between 1 and 6) of crossover events between the two sequences constrained to the homologous regions. We emitted two VCF entries for each NAHR event, the first replacing one *var* sequence with our new sequence, the other removing the second *var* gene from the progeny reference.

S5.3 Other variants

We simulated SNVs, short indels and inversions, multi-nucleotide polymorphisms. For the non-point mutations, we simulated a range of allele sizes up to 1,000 bp. To evaluate our capabilities in expansions or contractions of repetitive sequence, we scanned the parental reference assemblies for series of repeating sequence units up to length 6 bp, adding or removing units up to the maximum number of units present in the repeat. All events were added to the VCF used for draft reference sequence permutation.

S5.4 Read simulation

Reads were simulated using 76 bp reads, insert size of 250 bp, a mean read depth of 120x, and per-base error rate of 0.5%. All processing steps proceeded identically to the procedure specified in section S4.5.

S5.5 Evaluation

To evaluate performance of our mutation caller, we developed the `EvaluateCalls` module in Corticall. To overcome issues regarding different (but effectively equivalent) descriptions of alleles between the simulation VCF and the Corticall output VCF, we reused the reference permutation concept from S1.2. We permuted the indicated draft reference genomes with the simulated and called VCFs and evaluated local sequence equivalence between the two callsets. We emitted precision and recall metrics for each variant, and additional information regarding variants that had been incompletely assembled.

Bibliography

[1] S. F. Altschul, W. Gish, W. Miller, E. W. Myers, and D. J. Lipman. Basic local alignment search tool. *Journal of molecular biology*, 215(3):403–410, Oct. 1990.

[2] S. F. Altschul, W. Gish, W. Miller, E. W. Myers, and D. J. Lipman. Basic local alignment search tool. *Journal of molecular biology*, 215(3):403–410, Oct. 1990.

[3] C. Aurrecoechea, J. Brestelli, B. P. Brunk, J. Dommer, S. Fischer, B. Gajria, X. Gao, A. Gingle, G. Grant, O. S. Harb, M. Heiges, F. Innamorato, J. Iodice, J. C. Kissinger, E. Kraemer, W. Li, J. A. Miller, V. Nayak, C. Pennington, D. F. Pinney, D. S. Roos, C. Ross, C. J. Stoeckert, C. Treatman, and H. Wang. PlasmoDB: a functional genomic database for malaria parasites. *Nucleic acids research*, 37(Database):D539–D543, Jan. 2009.

[4] M. A. DePristo, E. Banks, R. Poplin, K. V. Garimella, J. R. Maguire, C. Hartl, A. A. Philippakis, G. del Angel, M. A. Rivas, M. Hanna, A. McKenna, T. J. Fennell, A. M. Kernytsky, A. Y. Sivachenko, K. Cibulskis, S. B. Gabriel, D. Altshuler, and M. J. Daly. A framework for variation discovery and genotyping using next-generation DNA sequencing data. *Nature genetics*, 43(5):491–498, May 2011.

[5] R. Durbin. *Biological Sequence Analysis*. Probabilistic Models of Proteins and Nucleic Acids. Cambridge University Press, Apr. 1998.

[6] M. J. Gardner, N. Hall, E. Fung, O. White, M. Berriman, R. W. Hyman, J. M. Carlton, A. Pain, K. E. Nelson, S. Bowman, I. T. Paulsen, K. James, J. A. Eisen, K. Rutherford, S. L. Salzberg, A. Craig, S. Kyes, M.-S. Chan, V. Nene, S. J. Shallom, B. Suh, J. Peterson, S. Angiuoli, M. Pertea, J. Allen, J. Selengut, D. Haft, M. W. Mather, A. B. Vaidya, D. M. A. Martin, A. H. Fairlamb, M. J. Fraunholz, D. S. Roos, S. A. Ralph, G. I. McFadden, L. M. Cummings, G. M. Subramanian, C. Mungall, J. C. Venter, D. J. Carucci, S. L. Hoffman, C. Newbold, R. W. Davis, C. M. Fraser, and B. Barrell. Genome sequence of the human malaria parasite Plasmodium falciparum. *Nature*, 419(6906):498–511, Oct. 2002.

[7] A. J. Gibbs and G. A. Mcintyre. The Diagram, a Method for Comparing Sequences. *European Journal of Biochemistry*, 16(1):1–11, Sept. 1970.

[8] K. Hayton, D. Gaur, A. Liu, J. Takahashi, B. Henschen, S. Singh, L. Lambert, T. Furuya, R. Bouttenot, M. Doll, F. Nawaz, J. Mu, L. Jiang, L. H. Miller, and T. E. Wellems. Erythrocyte Binding Protein PfRH5 Polymorphisms Determine Species-Specific Pathways of Plasmodium falciparum Invasion. *Cell Host & Microbe*, 4(1):40–51, Jan. 2008.

[9] Z. Iqbal, M. Caccamo, I. Turner, P. Flicek, and G. McVean. De novo assembly and genotyping of variants using colored de Bruijn graphs. *Nature genetics*, 44(2):226–232, Feb. 2012.

[10] I. Kozarewa, Z. Ning, M. A. Quail, M. J. Sanders, M. Berriman, and D. J. Turner. Amplification-free Illumina sequencing-library preparation facilitates improved mapping and assembly of (G+C)-biased genomes. *Nature methods*, 6(4):291–295, Mar. 2009.

[11] S. Kurtz, A. Phillippy, A. L. Delcher, M. Smoot, M. Shumway, C. Antonescu, and S. L. Salzberg. Versatile and open software for comparing large genomes. *Genome Biology*, 5(2):R12, Jan. 2004.

[12] H. Li. Aligning sequence reads, clone sequences and assembly contigs with BWA-MEM. *arXiv.org*, page arXiv:1303.3997, Mar. 2013.

[13] N. Li and M. Stephens. Modeling linkage disequilibrium and identifying recombination hotspots using single-nucleotide polymorphism data. *Genetics*, 165(4):2213–2233, Dec. 2003.

[14] E. K. C. D. S. C. P. A. K. P. D. G. M. Martine M Zilversmit. Hypervariable antigen genes in malaria have ancient roots. *BMC Evolutionary Biology*, 13:110, 2013.

[15] A. Miles, Z. Iqbal, P. Vauterin, R. Pearson, S. Campino, M. Theron, K. Gould, D. Mead, E. Drury, J. O'Brien, V. Ruano Rubio, B. MacInnis, J. Mwangi, U. Samarakoon, L. Ranford-Cartwright, M. Ferdig, K. Hayton, X.-Z. Su, T. Wellems, J. Rayner, G. McVean, and D. Kwiatkowski. Indels, structural variation, and recombination drive genomic diversity in Plasmodium falciparum. *Genome Research*, 26(9):1288–1299, Sept. 2016.

[16] A. Miles, Z. Iqbal, P. Vauterin, R. Pearson, S. Campino, M. Theron, K. Gould, D. Mead, E. Drury, J. O'Brien, V. R. Rubio, B. MacInnis, J. Mwangi, U. Samarakoon, L. Ranford-Cartwright, M. Ferdig, K. Hayton, X. Su, T. Wellems, J. Rayner, G. McVean, and D. Kwiatkowski. Genome variation and meiotic recombination in Plasmodium falciparum: insights from deep sequencing of genetic crosses. *bioRxiv*, page 024182, Aug. 2015.

[17] T. D. Otto, U. Böhme, M. Sanders, A. Reid, E. I. Bruske, C. W. Duffy, P. C. Bull, R. D. Pearson, A. Abdi, S. Dimonte, L. B. Stewart, S. Campino, M. Kekre, W. L. Hamilton, A. Claessens, S. K. Volkman, D. Ndiaye, A. Amambua-Ngwa, M. Diakité, R. M. Fairhurst, D. J. Conway, M. Franck, C. I. Newbold, and M. Berriman. Long read assemblies of geographically dispersed Plasmodium falciparum isolates reveal highly structured subtelomeres. *Wellcome Open Research*, 3:52, May 2018.

[18] T. D. Otto, G. P. Dillon, W. S. Degrave, and M. Berriman. RATT: Rapid Annotation Transfer Tool. *Nucleic acids research*, 39(9):e57–e57, May 2011.

[19] E. A. Ozer, J. P. Allen, and A. R. Hauser. Characterization of the core and accessory genomes of Pseudomonas aeruginosa using bioinformatic tools Spine and AGEnt. *BMC genomics*, 15(1):737, Dec. 2014.

[20] R. Poplin, V. Ruano Rubio, M. A. DePristo, T. J. Fennell, M. O. Carneiro, G. A. Van der Auwera, D. E. Kling, L. D. Gauthier, A. Levy-Moonshine, D. Roazen, K. Shakir, J. Thibault, S. Chandran, C. Whelan, M. Lek, S. Gabriel, M. J. Daly, B. Neale, D. G. MacArthur, and E. Banks. Scaling accurate genetic variant discovery to tens of thousands of samples. *bioRxiv*, page 201178, Nov. 2017.

[21] T. Rausch, T. Zichner, A. Schlattl, A. M. Stütz, V. Benes, and J. O. Korbel. DELLY: structural variant discovery by integrated paired-end and split-read analysis. 28(18):i333–i339, Sept. 2012.

[22] J. M. Sá, S. R. Kaslow, M. A. Krause, V. A. Melendez-Muniz, R. E. Salzman, W. A. Kite, M. Zhang, R. R. M. Barros, J. Mu, P. K. Han, J. P. Mershon, C. E. Figan, R. L. Caleon, R. S. Rahman, T. J. Gibson, C. Amaratunga, E. P. Nishiguchi, K. F. Breglio, T. M. Engels, S. Velmurugan, S. Ricklefs, J. Straimer, N. F. Gnadig, B. Deng, A. Liu, A. Diouf, K. Miura, G. S. Tullo, R. T. Eastman, S. Chakravarty, E. R. James, K. Udenze, S. Li, D. E. Sturdevant, R. W. Gwadz, S. F. Porcella, C. A. Long, D. A. Fidock, M. L. Thomas, M. P. Fay, B. K. L. Sim, S. L. Hoffman, J. H. Adams, R. M. Fairhurst, X.-z. Su, and T. E. Wellems. Artemisinin resistance phenotypes and K13 inheritance in a Plasmodium falciparum cross and Aotus model. *Proceedings of the National Academy of Sciences*, pages 1–6, Nov. 2018.

[23] A. Smit, R. Hubley, and P. Green. *RepeatMasker Open-4.0*, 2013.

[24] S. Steinbiss, F. Silva-Franco, B. Brunk, B. Foth, C. Hertz-Fowler, M. Berriman, and T. D. Otto. Companion: a web server for annotation and analysis of parasite genomes. *Nucleic acids research*, 44(W1):W29–W34, July 2016.

[25] W. Trager and J. B. Jensen. Human malaria parasites in continuous culture. *sciencemag.org*, 193(4254):673–675, Aug. 1976.

[26] I. Turner, K. V. Garimella, Z. Iqbal, and G. McVean. Integrating long-range connectivity information into de Bruijn graphs. *bioRxiv*, page 147777, June 2017.

[27] I. Turner, K. V. Garimella, Z. Iqbal, and G. McVean. Integrating long-range connectivity information into de Bruijn graphs. *Bioinformatics (Oxford, England)*, 34(15):2556–2565, Aug. 2018.

[28] D. Walliker, I. Quakyi, T. Wellems, T. McCutchan, A. Szarfman, W. London, L. Corcoran, T. Burkot, and R. Carter. Genetic analysis of the human malaria parasite Plasmodium falciparum. 236(4809):1661–1666, June 1987.

[29] T. E. Wellems, L. J. Panton, I. Y. Gluzman, V. E. do Rosario, R. W. Gwadz, A. Walker-Jonah, and D. J. Krogstad. Chloroquine resistance not linked to mdr-like genes in a Plasmodium falciparum cross. *Nature*, 345(6272):253–255, May 1990.
