# Supplementary material for: Detection of simple and complex de novo mutations with multiple reference sequences
Source: Genome Res. 2020 Aug;30(8):1154–69. doi: 10.1101/gr.255505.119 (PMC7462078; doi:10.1101/gr.255505.119)
Supplement: Supplemental Material [file supp_gr.255505.119_Supplemental_Code.tar.zip › Supplemental_Code.tar.gz!/Corticall-0.5/public/html/GraphContext.html]

Contig viewer


# Graph context

Ready.
